# Supplementary material for: Prevalence and identification of anxiety disorders in pregnancy: the diagnostic accuracy of the two-item Generalised Anxiety Disorder scale (GAD-2)
Source: BMJ Open. 2018 Sep 5;8(9):e023766. doi: 10.1136/bmjopen-2018-023766 (PMC6129087; doi:10.1136/bmjopen-2018-023766)
Supplement: Supplementary file 1 [file bmjopen-2018-023766supp001.pdf]

Online supplementary file 1

Table showing characteristics of wider base population and study population

	Base population*	WENDY study sample N=545	Sample with GAD-2 data N=528
**Age (years)**	Mean: 31.67 Range: 14 – 52	Mean: 32.85 range: 16 – 47.5	Mean: 32.84 Range: 16 – 47.5
16 – 19	232 (2%)	8 (1%)	7 (1%)
20 – 29	3048 (30%)	150 (28%)	144 (27%)
30 – 39	6240 (61%)	341 (63%)	336 (64%)
40+	705 (7%)	46 (8%)	41 (8%)
**Ethnicity**			
White	4914 (51%)	284 (52%)	279 (53%)
Black/ Caribbean	3162 (33%)	177 (32%)	169 (32%)
Asian/Asian British	594 (6%)	25 (5%)	22 (4%)
Mixed/Multiple ethnicity	308 (3%)	23 (4%)	22 (4%)
Other	646 (7%)	36 (7%)	36 (7%)
**Other children**			
None	5077 (50%)	271 (50%)	264 (50%)
1	3209 (31%)	175 (32%)	167 (32%)
2 or more	1939 (19%)	99 (18%)	97 (18%)

*Base population covers women seen from 1.11.2014 - 30.6.2016, missing data not included.
