# Supplementary material for: Prevalence and identification of anxiety disorders in pregnancy: the diagnostic accuracy of the two-item Generalised Anxiety Disorder scale (GAD-2)
Source: BMJ Open. 2018 Sep 5;8(9):e023766. doi: 10.1136/bmjopen-2018-023766 (PMC6129087; doi:10.1136/bmjopen-2018-023766)
Supplement: Supplementary file 8 [file bmjopen-2018-023766supp008.pdf]

Online supplementary file 8

Table of weighted population prevalence's for calculations of sensitivity and specificity for any anxiety disorder (excluding PTSD and OCD)

	**GAD-2 (<3) negative**	**GAD-2 (≥3) positive**
**No anxiety**	7536<br>88% (83 – 91%)	805.5<br>69 (54 – 80%)
**SCID any anxiety**	1039<br>12% (9 – 17%)	368.8<br>31 (20 – 46%)
	**GAD-2 (Yes/No) negative**	**GAD-2 (Yes/No) positive**
**No anxiety**	5187<br>92% (87 – 95%)	3155<br>77% (69 – 83%)
**SCID any anxiety**	446.5<br>8% (5 – 13%)	961.3<br>23% (17 – 31%)
