# Supplementary material for: Prevalence and identification of anxiety disorders in pregnancy: the diagnostic accuracy of the two-item Generalised Anxiety Disorder scale (GAD-2)
Source: BMJ Open. 2018 Sep 5;8(9):e023766. doi: 10.1136/bmjopen-2018-023766 (PMC6129087; doi:10.1136/bmjopen-2018-023766)
Supplement: Supplementary file 9 [file bmjopen-2018-023766supp009.pdf]

**Online supplementary file 9**

**Table of weighted population prevalence's for calculations of sensitivity and specificity for any anxiety disorder (including PTSD and OCD) (missing PTSD data considered as PTSD cases)**

| | **GAD-2 (<3) negative** | **GAD-2 (≥3) positive** |
|---|---|---|
| **No anxiety** | 7268<br>85% (80 – 89%) | 723.1<br>62% (47 – 74%) |
| **SCID any anxiety** | 1307<br>15% (11 – 20%) | 451.3<br>38% (26 – 53%) |
| | | |
| | **GAD-2 (Yes/No) negative** | **GAD-2 (Yes/No) positive** |
| **No anxiety** | 5110<br>91% (85 – 94%) | 2881<br>70% (62 – 77%) |
| **SCID any anxiety** | 523<br>9% (6 – 15%) | 1235<br>30% (23 – 38%) |

**Table of weighted population prevalence's for calculations of sensitivity and specificity for any anxiety disorder (including PTSD and OCD) (missing PTSD data considered as not PTSD cases)**

| | **GAD-2 (<3) negative** | **GAD-2 (≥3) positive** |
|---|---|---|
| **No anxiety** | 7450<br>87% (82 – 90%) | 770.8<br>66% (51 – 78%) |
| **SCID any anxiety** | 1125<br>13% (10 – 18%) | 403.6<br>34% (22 – 49%) |
| | | |
| | **GAD-2 (Yes/No) negative** | **GAD-2 (Yes/No) positive** |
| **No anxiety** | 5181<br>92%(87 – 95%) | 3040<br>74% (66 – 80%) |
| **SCID any anxiety** | 452.8<br>8% (5 – 13%) | 1076<br>26 (20 – 34%) |
