# Supplementary material for: Prevalence and identification of anxiety disorders in pregnancy: the diagnostic accuracy of the two-item Generalised Anxiety Disorder scale (GAD-2)
Source: BMJ Open. 2018 Sep 5;8(9):e023766. doi: 10.1136/bmjopen-2018-023766 (PMC6129087; doi:10.1136/bmjopen-2018-023766)
Supplement: Supplementary file 2 [file bmjopen-2018-023766supp002.pdf]

Online supplementary file 2

Table of weighted population prevalence for calculations of sensitivity and specificity for GAD including count and proportion % (95% CI)

	**GAD-2 (<3) negative**	**GAD-2 (≥3) positive**
**No GAD**	8439<br>98% (97 – 99%)	872.2<br>74% (60 – 85%)
**SCID GAD**	136.5<br>2% (1 – 3%)	302.2<br>26% (15 – 40%)
	**GAD-2 (Yes/No) negative**	**GAD-2 (Yes/No) positive**
**No GAD**	5633<br>100%	3678<br>89% (84 – 93%)
**SCID GAD**	0<br>0%	438.7<br>11% (7 – 16%)
