# Supplementary figures and images for: Prevalence and identification of anxiety disorders in pregnancy: the diagnostic accuracy of the two-item Generalised Anxiety Disorder scale (GAD-2)
Source: BMJ Open. 2018 Sep 5;8(9):e023766. doi: 10.1136/bmjopen-2018-023766 (PMC6129087; doi:10.1136/bmjopen-2018-023766)

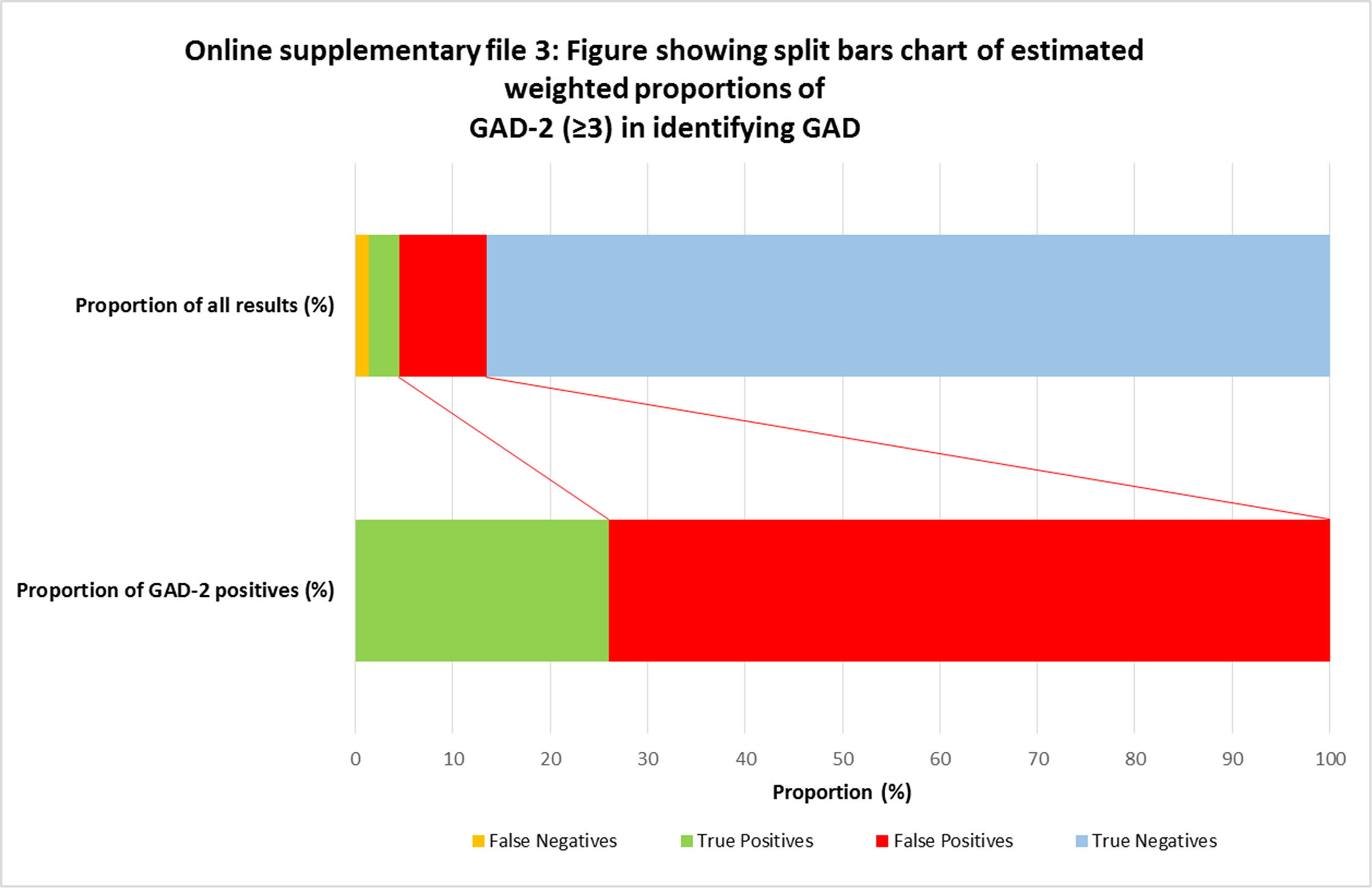

Supplement: Supplementary file 3 [file bmjopen-2018-023766supp003.jpg]

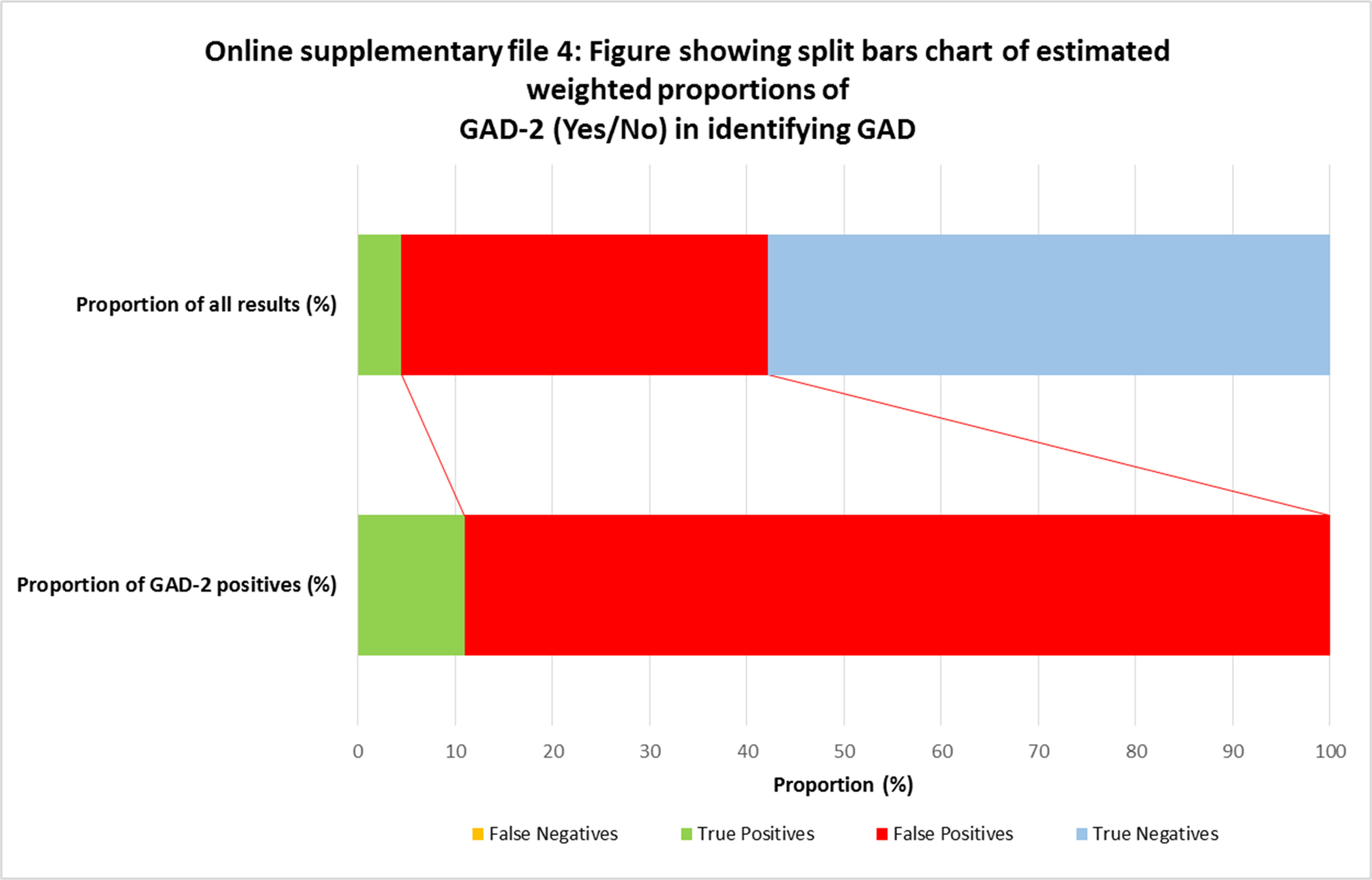

Supplement: Supplementary file 4 [file bmjopen-2018-023766supp004.jpg]

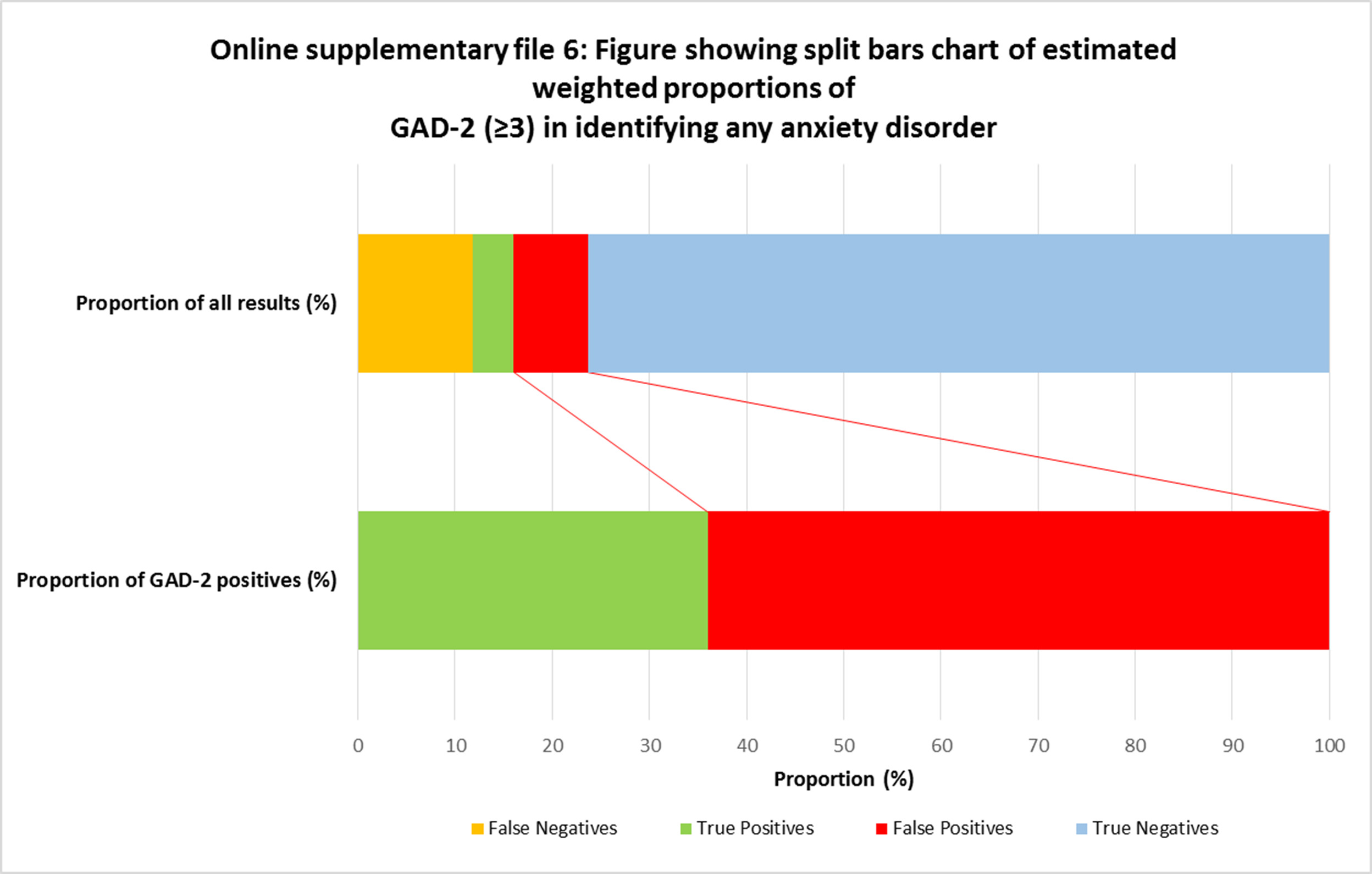

Supplement: Supplementary file 6 [file bmjopen-2018-023766supp006.jpg]

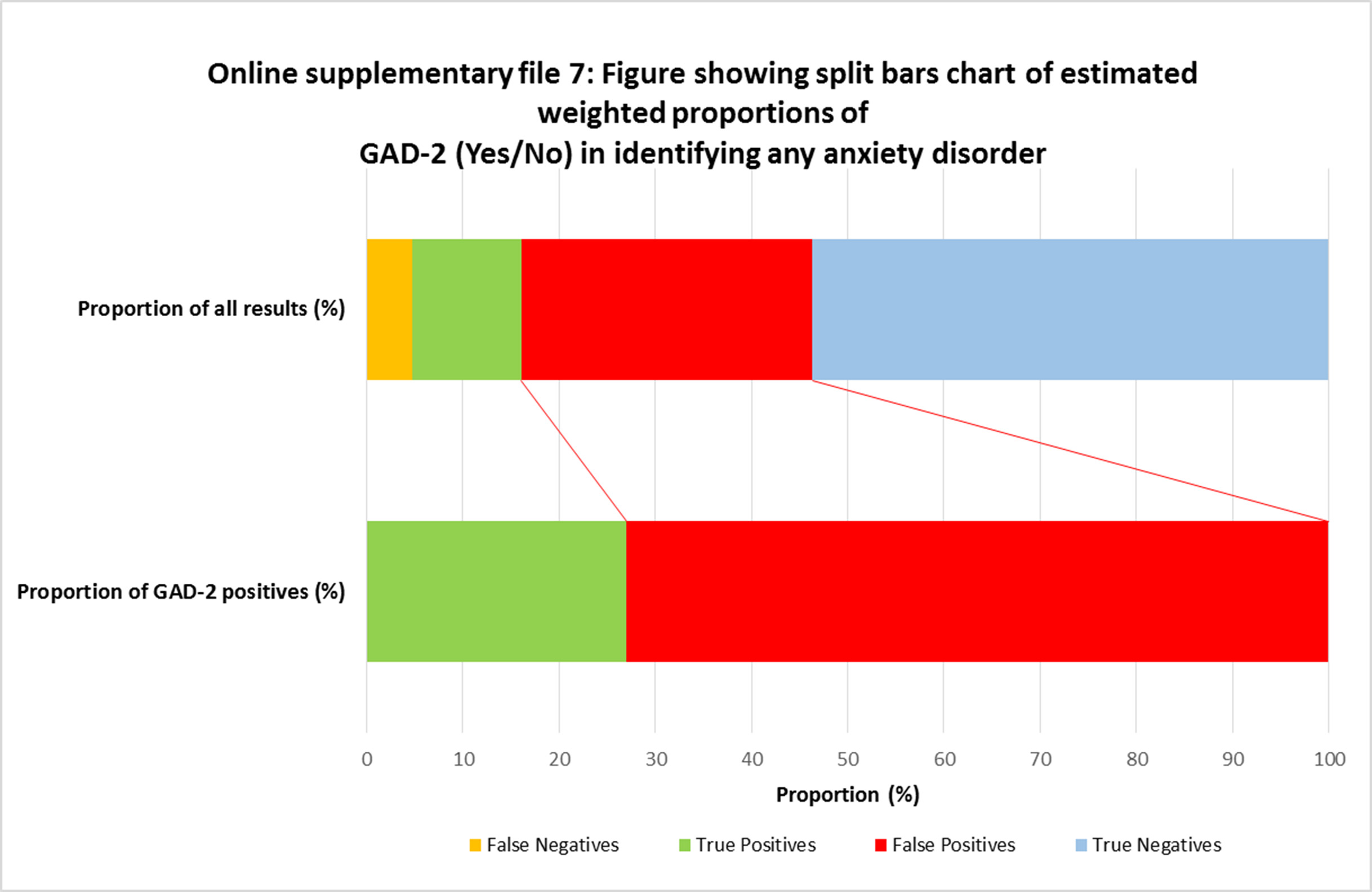

Supplement: Supplementary file 7 [file bmjopen-2018-023766supp007.jpg]
