# Supplementary material for: Prevalence and identification of anxiety disorders in pregnancy: the diagnostic accuracy of the two-item Generalised Anxiety Disorder scale (GAD-2)
Source: BMJ Open. 2018 Sep 5;8(9):e023766. doi: 10.1136/bmjopen-2018-023766 (PMC6129087; doi:10.1136/bmjopen-2018-023766)
Supplement: Supplementary file 5 [file bmjopen-2018-023766supp005.pdf]

Online supplementary file 5

Table of weighted population prevalence for calculations of sensitivity and specificity for any anxiety disorder (including PTSD and OCD) including count and proportion % (95% CI)

	**GAD-2 (<3) negative**	**GAD-2 (≥3) positive**
**No anxiety**	7268<br>87% (82 – 90%)	723.1<br>64% (49 – 77%)
**SCID any anxiety**	1125<br>13% (10 – 18%)	403.6<br>36% (23 – 51%)
	**GAD-2 (Yes/No) negative**	**GAD-2 (Yes/No) positive**
**No anxiety**	5110<br>92% (87 – 95%)	2881<br>73% (65 – 80%)
**SCID any anxiety**	452.8<br>8% (5 – 13%)	1076<br>27% (20 – 35%)
