# Supplementary material for: Prevalence and identification of anxiety disorders in pregnancy: the diagnostic accuracy of the two-item Generalised Anxiety Disorder scale (GAD-2)
Source: BMJ Open. 2018 Sep 5;8(9):e023766. doi: 10.1136/bmjopen-2018-023766 (PMC6129087; doi:10.1136/bmjopen-2018-023766)
Supplement: Author's manuscript [file bmjopen-2018-023766.draft_revisions.pdf]

BMJ Open

BMJ Open is committed to open peer review. As part of this commitment we make the peer review history of every article we publish publicly available.

When an article is published we post the peer reviewers' comments and the authors' responses online. We also post the versions of the paper that were used during peer review. These are the versions that the peer review comments apply to.

The versions of the paper that follow are the versions that were submitted during the peer review process. They are not the versions of record or the final published versions. They should not be cited or distributed as the published version of this manuscript.

BMJ Open is an open access journal and the full, final, typeset and author-corrected version of record of the manuscript is available on our site with no access controls, subscription charges or pay-per-view fees (http://bmjopen.bmj.com).

If you have any questions on BMJ Open's open peer review process please email info.bmjopen@bmj.com

BMJ Open

The prevalence and identification of anxiety disorders in pregnancy: the diagnostic accuracy of the two item Generalized Anxiety Disorder scale (GAD-2)

Journal:	*BMJ Open*
Manuscript ID	bmjopen-2018-023766
Article Type:	Research
Date Submitted by the Author:	26-Apr-2018
Complete List of Authors:	Nath, Selina; Institute of Psychiatry, Psychology & Neuroscience, King's College London, Health Service and Population Research Department<br>Ryan, Elizabeth ; King's College London, Biostatistics Department<br>Trevillion, Kylee; Kings College London, Health Service and Population Research Department<br>Bick, Debra; Kings College London, Florence Nightingale School of Nursing and Midwifery<br>Demilew, Jill; King's Health Partners, Women's Health Academic Centre<br>Milgrom, Jeannette; University of Melbourne Melbourne School of Psychological Sciences, School of Psychological Sciences<br>Pickles, Andrew; Kings College London<br>Howard, Louise; institute of psychiatry, health service and population research
Keywords:	Maternal, Anxiety disorders < PSYCHIATRY, GAD, GAD-2, Diagnostic accuracy

SCHOLARONE™
Manuscripts

Title: The prevalence and identification of anxiety disorders in pregnancy: the diagnostic accuracy of the two item Generalized Anxiety Disorder scale (GAD-2)

Corresponding Author:

Selina Nath, selina.nath@kcl.ac.uk

Section of Women's Mental Health, Health Service and Population Research Department, Institute of Psychiatry, Psychology & Neuroscience, King's College London, De Crespigny Park, London SE5 8AF, UK.

Telephone number: +44(0)20 7848 5009

Authors:

Dr Selina Nath [1] (Postdoctoral Researcher)

Dr Elizabeth G Ryan [2,7] (Clinical Trials Statistician)

Dr Kylee Trevillion [1] (Programme manager)

Prof Debra Bick [3] (Professor of Evidence Based Midwifery)

Jill Demilew [4] (Consultant Midwife at King's College Hospital)

Prof Jeannette Milgrom [5] (Professor of Psychology)

Prof Andrew Pickles [2] (Professor of Biostatistics and Psychological Methods)

Prof Louise M Howard [1,3,6] (Professor of Women's Mental Health)

[1] Section of Women's Mental Health, Health Service and Population Research Department, Institute of Psychiatry, Psychology & Neuroscience, King's College London, De Crespigny Park, London SE5 8AF, UK.

[2] Biostatistics and Health Informatics Department, Institute of Psychiatry, Psychology and Neuroscience, King's College London, De Crespigny Park, London SE5 8AF, UK.

[3] Department of Women and Children's Health, School of Life Course Sciences, Faculty of Life Sciences and Medicine, King's College London, Westminster Bridge Road, London, SE1 7EH, UK.

[4] Women's Health, King's College Hospital NHS Foundation Trust, Denmark Hill, London SE5 8RS

[5] Parent-Infant Research Institute (PIRI®), Austin Health, Melbourne School of Psychological Sciences, University of Melbourne, VIC 3010, Australia.

[6] South London and Maudsley NHS Foundation Trust, London SW2 1NU, UK.

[7] Warwick Clinical Trials Unit, Warwick Medical School, University of Warwick, Coventry, CV4 7AL, UK.

Word count: 3,813 words

For peer review only - http://bmjopen.bmj.com/site/about/guidelines.xhtml

ABSTRACT

Objective: To estimate the population prevalence of anxiety disorders during pregnancy and investigate the diagnostic accuracy of the GAD-2 for a) Generalised Anxiety Disorder and b) any anxiety disorder.

Design: Cross-sectional survey using a stratified sampling design. Sampling weights were used in the analysis to adjust for the bias introduced by the stratified sampling.

Setting: Inner-city maternity service, South London.

Participants: 545 pregnant women were interviewed after their first antenatal appointment; 528 provided answers on the GAD-2 questions.

Main outcome measures: Diagnosis generated by the Structured Clinical Interview for DSM-IV (SCID).

Results: Population prevalence of anxiety disorders was 17% (95%CI: 12 to 21%): 5% (95%CI: 3 to 6%) for Generalised Anxiety Disorder, 4% (95%CI: 2 to 6%) social phobia, 8% (95%CI: 5 to 11%) specific phobia and 2% (95%CI:1 to 4%) for obsessive-compulsive disorder. Post-Traumatic Stress Disorder prevalence was unclear due to higher levels of reluctance to respond to PTSD interview questions but sensitivity analyses suggest population prevalence maybe up to 4% (95%CI: 2 to 6%). Weighted sensitivity of GAD-2 for Generalised Anxiety Disorder (cut-off ≥3) was 69%, specificity 91%, positive predictive value 26%, negative predictive value 98% and Likelihood Ratio 7.35. For any anxiety disorder the weighted sensitivity was 26%, specificity 91%, positive predictive value 36%, negative predictive value 87%, and Likelihood Ratio 2.92.

Conclusions: Anxiety disorders are common but the GAD-2 generates many false positives and may therefore be unhelpful in maternity services.

Key words: Anxiety, pregnancy, prevalence, GAD-2, diagnostic accuracy

Registration: UKCRN ID: 16403 (Previously at http://public.ukcrn.org.uk/search/StudyDetail.aspx?StudyID=16403; now at https:/cpms.nihr.ac.uk).

For peer review only - http://bmjopen.bmj.com/site/about/guidelines.xhtml

ARTICLE SUMMARY:

STRENGTHS AND LIMITATIONS OF THIS STUDY:

- This study investigates the effectiveness of the Generalized Anxiety Disorder scale (GAD-2) in identifying antenatal anxiety disorders – a method suggested by NICE (CG192 2014) on the basis of expert consensus rather than research evidence.

- We recruited a representative sample of women using an efficient and robust sampling design which facilitated the estimation of population prevalence's.

- We used a gold standard diagnostic interview.

- This research was limited to recruitment from a single maternity site in London, though the single South London maternity site utilised was a very ethnically and socioeconomically diverse population.

For peer review only

For peer review only - http://bmjopen.bmj.com/site/about/guidelines.xhtml

INTRODUCTION

Anxiety disorders are more common in women than men,[1 2] and the perinatal period (i.e. pregnancy and the year after birth) has been reported as a particularly vulnerable time for the onset or relapse of anxiety disorders in women.[3 4] Antenatal anxiety disorders have been associated with adverse pregnancy outcomes, including preterm birth, low birth weight, lower Apgar scores, postpartum anxiety and depression, and adverse child developmental outcomes[5-7] including difficult temperament, increased sleep problems, bonding/attachment problems, and poorer emotional, behavioural and cognitive development.[5-10] A recent systematic review and meta-analysis reported a pooled prevalence of 4% (95%CI: 2 to 6%; 10 studies with pooled n=6910) for Generalised Anxiety Disorder (GAD) and 15% (95%CI: 9 to 21%; 9 studies with pooled n=4648) for anxiety disorders during pregnancy based on studies conducted outside of the UK where diagnostic clinical interviews were utilised, and 23% when using cut-offs on validated self-report questionnaires.[11] Anxiety disorders are treatable, so early detection and treatment during the antenatal period, when women are in regular contact with healthcare professionals could prevent adverse outcomes.[12-14] However, despite regular contact with healthcare professionals during pregnancy, antenatal mental disorders are often undetected and untreated.[15 16]

NICE (CG192; 2014) suggested that maternity professionals could consider the use of the 2-item Generalized Anxiety Disorders-2 tool (GAD-2) to identify anxiety disorders during pregnancy and after birth, though also highlighted the lack of evidence on the use of the GAD-2 in early pregnancy. The recommendation was therefore driven by concern about the high prevalence of anxiety disorders (NICE 2014).[12 17] This extends the focus of early intervention from a previous emphasis on identification of perinatal depression to other perinatal mental disorders, including comorbid conditions. Outside of the perinatal period, a systematic review and meta-analysis[18] of studies in men and women reported that the GAD-2 (using a cut-off of ≥3) showed fairly high pooled sensitivity 0.76 (95%CI: 0.55 to 0.89), and pooled specificity 0.81 (95%CI: 0.60 to 0.92) for GAD (5 studies with pooled n=1987). For detecting any anxiety disorder, the systematic review[18] reported a moderate sensitivity (range: 0.65 to 0.72) and unclear specificity (range: 0.39 to 0.92) (3 studies, N=1225). The diagnostic accuracy of the GAD-2 questions in identifying anxiety disorders during early pregnancy remains unstudied. As women in early pregnancy are likely to have many anxieties (e.g. over the viability of the pregnancy, decisional conflict over unplanned pregnancies), there may be high rates of “false positives” when using the GAD-2 in pregnancy which could result in inappropriate referrals to mental health services.

For peer review only - http://bmjopen.bmj.com/site/about/guidelines.xhtml

We therefore aimed to investigate:

1) The UK prevalence of Generalised Anxiety Disorder (GAD), other anxiety disorders (including panic disorder, agoraphobia without panic disorder, social phobia, specific phobia, Obsessive-Compulsive Disorder (OCD), and Post-Traumatic Stress Disorder (PTSD)) and comorbidity with other mental disorders during early pregnancy.
2) The sensitivity, specificity, Positive Predictive Value (PPV), Negative Predictive Value (NPV), positive likelihood ratio (LR+) and negative likelihood ratio (LR-) of the GAD-2 screening questions (on a Likert scale) compared with a gold standard diagnostic interview (DSM-IV)[19] for identifying GAD, and for identifying any anxiety disorders (including panic disorder, agoraphobia without panic disorder, social phobia, specific phobia, Obsessive-Compulsive Disorder (OCD), Post-Traumatic Stress Disorder (PTSD) and GAD) during early pregnancy. As DSM-V [20] no longer categorises OCD or PTSD as anxiety disorders, we also investigated diagnostic accuracy for any anxiety disorders by excluding OCD and PTSD.
3) How the sensitivity, specificity, PPV, NPV, LR+ and LR- change when the GAD-2 is scored and categorised as a Yes (cut-off 1 or more) verses No (score of 0) response instead of the conventional cut-off of 3 or more (which could be asked by midwives at the same time as the two depression screening questions).

METHODS

Study design and participants

The WEll-being in pregNancy stuDY (WENDY study) was a cross-sectional survey that recruited women from an inner city maternity service in South-east London using a sampling design stratified according to answering positive or negative (saying yes or no respectively) on either of the two Whooley questions which are routinely asked by midwives as a mental health screen during the first antenatal booking appointment ("During the past month have you often been bothered by feeling down, depressed, or hopeless?"; "During the past month have you often been bothered by having little interest or pleasure in doing things?"). A random sample of Whooley negative and all Whooley positive women were invited to participate.

For peer review only - http://bmjopen.bmj.com/site/about/guidelines.xhtml

Exclusion criteria were women under 16 years of age, women who declined to answer Whooley questions, those who had a termination or miscarriage prior to baseline interview or had already attended for their maternity booking appointment elsewhere in the UK. Eligible pregnant women who agreed to participate were recruited into the study as soon as possible after their first antenatal booking appointment, within a maximum of three weeks from the original booking appointment. Data collection of the index test (GAD-2 measure) and reference test (the gold standard diagnostic interview) were performed during the research interview, after written informed consent was obtained. Language interpreters were used where needed. For further details and power calculation of the WENDY study see Howard et al. (2018).[21] Sample size of the current analysis was determined by the number of women with available data on the index test (GAD-2). Figure 1 shows flow chart of women through the WENDY study and those used for the current analysis.

[INSERT FIGURE 1 HERE]

Research Measures

Generalized Anxiety Disorder Scale (GAD-2): The GAD-2 is a sub-scale of the Generalized Anxiety Disorder Scale GAD-7 measure[22] and is a two-item self-report screen completed by women during the research interview. The questions include "Over the last two weeks, how often have you been bothered by any of the following problems? 1) Feeling nervous, anxious or on edge; 2) Not being able to stop or control worrying"). Answers are given on a Likert scale (not at all=0, several days=1, more than half the days=2 and nearly everyday=3). Scores range from 0 - 6, with a cut-off score of 3 or more indicative of anxiety symptoms.[23] The GAD-2 has also been used where YES to either question categorises an individual as a positive screen (used in clinical practice),[24] thus a score of 1 or more would indicate a possible YES answer. In this study, women's responses to the GAD-2 questions were categorised into the following groups:

1) GAD-2 (≥3): GAD-2 positive if they scored 3 or more (conventional scoring method), GAD-2 negatives if they scored less than 3.
2) GAD-2 (Yes/No): GAD-2 positive if they scored 1 or more (indicating a "yes" to either question) which is used more in clinical practice, GAD-2 negative if scoring 0 (indicating "no" to both questions).

For peer review only - http://bmjopen.bmj.com/site/about/guidelines.xhtml

Anxiety disorders and comorbid disorders

The Structured Clinical Interview for DSM-IV Axis I Disorders (SCID-I): the SCID is a researcher-administered, semi-structured, and gold standard diagnostic interview consisting of standardised questions that correspond to each DSM-IV Axis I criteria.[19] We used the mood and anxiety disorders modules to generate diagnosis of depression, generalised anxiety disorder, panic disorder, agoraphobia without panic disorder, social phobia, specific phobia, obsessive-compulsive disorder, and post-traumatic stress disorder. Consensus on diagnosis was achieved during researcher's weekly meetings with LH. Although clinical information and GAD-2 data were available at the time of these meetings, agreement on diagnosis was reached using responses given by women during the SCID interviews (i.e. the GAD-2 items were not used to determine diagnosis of anxiety disorders).

As DSM-V[20] does not categorise OCD or PTSD as anxiety disorders and as the research version of the SCID for DSM-V was released after the start of the current study, we also carried out analyses for any anxiety disorders according to those included in DSM-V as anxiety disorders, i.e. excluding OCD and PTSD. We also used the eating disorder module to generate diagnosis for eating disorders including anorexia nervosa (including atypical), bulimia nervosa, binge eating disorder, purging disorder and other eating disorder.

Patient involvement

The development of the WENDY study, outcome measures, grant application, and study protocol were informed by our patient and carer advisory group. Meetings were held every few months to discuss the WENDY study and other related studies within a programme of work funded by NIHR (https://www.kcl.ac.uk/ioppn/depts/hspr/research/CEPH/wmh/projects/A-Z/esmi.aspx). The patient advisory group includes women with a range of mental disorders and have interest in our study programme. We circulated the results and draft manuscript (see acknowledgements) to members of the group for comments. Patients were not involved in the recruitment to and conduct of the study.

Statistical analysis

Data were managed and analysed using Stata v.15.[25] Sampling weights were used to adjust for the oversampling of Whooley positive women (See Howard et al, 2018)[26] in all analyses apart from examining differences in socio-demographics between GAD-2 positives and GAD-2 negatives. The population prevalence of anxiety disorders and comorbid disorders were estimated based on weighted

For peer review only - http://bmjopen.bmj.com/site/about/guidelines.xhtml

diagnostic interview responses (using Stata's 'svy' command). Bootstrap re-sampling of the weighted estimators was used for calculation of confidence intervals.

As pre-specified in our analysis plan, the weighted rates of "true positive", "false positives", "true negatives" and "false negatives" were tabulated for GAD, any anxiety disorder (including PTSD & OCD) and any anxiety disorder (without PTSD or OCD). Using these values, the sensitivity, specificity, PPV, NPV and likelihood ratios (positive and negative) were calculated.

Missing data

Of the 545 participants, 24 (4%) women had some SCID missing data: one participant on GAD and all eating disorders; one participant on agoraphobia, specific phobia, and PTSD; one participant on mixed anxiety and depression, Borderline Personality disorder and PTSD; one participant on hypomanic, manic, current major depressive disorder and bipolar I & II; one participant on all eating disorders; and 19 participants on the PTSD module. List-wise deletion (performed in Stata) was used to calculate frequencies of SCID disorders in the study sample.

To calculate population prevalence of the SCID disorders, missing observations in the SCID items were accounted for by using inverse probability weights that incorporated the Whooley sampling, as well as variables that were significant in predicting missingness of SCID responses (for full details of weightings and analysis strategy see Howard et al, 2018).[26] Due to the large numbers of women who declined to respond to questions on the PTSD module, we carried out a sensitivity analysis for the prevalence estimate of PTSD in which we first assumed that all missing data were actually cases of PTSD, and then assumed that all missing data were not cases of PTSD.

Seventeen participants (3%) had missing GAD-2 data (15 for both questions, 1 for GAD-2 question 1 and 1 for GAD-2 question 2). No imputation was performed for women with missing data on GAD-2 items because missing data for one question would mean 50% data missing. These were therefore treated as missing observations and only women with complete data on both GAD-2 questions were used in the analyses to investigate sensitivity and specificity (n=528). Out of 528 women with complete data on the GAD-2 questions, there was missing data on the following SCID anxiety modules: one participant had missing data on PTSD, agoraphobia and specific phobia and 18 participants had missing data on the PTSD module.

For peer review only - http://bmjopen.bmj.com/site/about/guidelines.xhtml

Ethics approval: The research was approved by the National Research Ethics Service, London Committee - Camberwell St Giles (ref no 14/LO/0075). Participants were provided with study information sheets which was fully explained to them, had the opportunity to ask questions and gave informed consent prior to taking part the study. No adverse events occurred for women taking part in the study during the research interview. Where risk (e.g. significant suicidality, safeguarding issues) was identified during the research interview, researchers discussed this with the study PI and the woman's midwife and/or GP were informed, following consent to information sharing by the study participant (all women were aware this occurred and consented to this).

RESULTS

Sample characteristics

Between 10/11/14 and 30/06/16, 10004 women attended their initial antenatal booking appointment with a midwife at the study site. Of these women, 41 did not have Whooley answers recorded so the base population consisted of 9963 women. The total number of eligible women recruited into the WENDY study was 545 women. This sample was similar to the base population on sociodemographic factors such as age, ethnicity and number of children (see Howard, et al. [21]). Of the total number of women recruited into the WENDY study, 528 (97%) provided answers to the GAD-2 questions (see Figure 1). Sociodemographic characteristics (age, ethnicity and number of children) of women that provided GAD-2 answers were similar to the rest of the WENDY sample and wider base population (see online supplementary file 1). There were 119 (23%) GAD-2(≥3) positives and 409 (77%) GAD-2 (<3) negatives within our study sample, where GAD-2 (≥3) positive was defined as reporting a total score of 3 or more on the GAD-2 questions (see Table 1). Compared to GAD-2 negatives, GAD-2 positives were more likely to be single, have lower levels of education, be unemployed or not working due to illness, have a lower income, and have an unplanned pregnancy. When scoring for GAD-2 positives was defined as scoring 1 or more, there were 226 (43%) GAD-2 (Yes/No) positives and 302 (57%) GAD-2 (Yes/No) negatives within our study sample.

[INSERT TABLE 1 HERE]

For peer review only - http://bmjopen.bmj.com/site/about/guidelines.xhtml

SCID Anxiety disorder prevalence and comorbidity

Using weighted estimates, the population prevalence was estimated as 17% (95%CI: 12 to 21%) for any SCID anxiety disorder (or 15% (95%CI: 11 to 19%) for any SCID anxiety disorder excluding PTSD and OCD, as in DSM-V). Specifically, there was an estimated population prevalence of 5% (95%CI: 3 to 6%) for GAD, 4% (95%CI: 2 to 6%) for social phobia, 8% (95%CI: 5 to 11%) for specific phobia, 0.2% (95%CI: 0.03 to 0.3%) for panic disorder, 0.4% (95%CI: 0 to 2%) for agoraphobia, 2% (95%CI: 1 to 4%) for OCD, and 0.8% (95%CI: 0 to 1%) for PTSD. As missing data were particularly common for the PTSD module, a sensitivity analysis demonstrated that when all women with missing data are assumed to have PTSD, the population prevalence estimate was 4% (95%CI: 2 to 6%). When all women with missing data are assumed not to have PTSD, the population prevalence estimate was 0.8% (95%CI: 0.1 to 1.4%). Of note, of the women who declined to respond to the PTSD questions, 8 had already disclosed severe trauma earlier during the research interview which were related to being physically abused, sexual abuse/rape and witnessing violence.

The estimated population prevalence of comorbid depression with any anxiety disorder was 5% (95%CI: 2 to 7%) (or 4% (95%CI: 2 to 6%) for comorbid depression and any anxiety disorder excluding PTSD and OCD). Comorbid depression and GAD was estimated as 2% (95%CI: 1 to 3%). Please see Table 2 for unweighted count (%) and weighted estimates of population prevalence (%) of comorbidity between SCID depression and all anxiety disorders (including PTSD and OCD).

[INSERT TABLE 2 HERE]

Diagnostic accuracy of the GAD-2 screening questions in identifying GAD

SCID GAD was found in 23 (6%) of GAD-2 (<3) negative and 35 (29%) of GAD-2 (≥3) positive women. After adjusting for weights, GAD was estimated to occur in 136.5 (2%, 95%CI: 1 to 3%) of GAD-2 (<3) negatives and 302.2 (26%, 95%CI: 15 to 40%) of GAD-2 (≥3) positives. 8439 (98%, 95%CI: 97 to 99%) GAD-2 (<3) negatives and 872.2 (74%, 95%CI: 60 to 85%) GAD-2 (≥3) positives did not meet criteria for SCID GAD (see online supplementary files 2 & 3 for cross-tabulation and proportions). Weighted sensitivity was 0.69, specificity 0.91, PPV 0.26, NPV 0.98, LR+ 7.35 and LR- 0.34. When scoring of the GAD-2 was changed to ≥1 (Yes/No response), 58 (19%) of GAD-2 (Yes/No) positive women met criteria

For peer review only - http://bmjopen.bmj.com/site/about/guidelines.xhtml

for SCID GAD, whereas there were no GAD-2 (Yes/No) negative women that met criteria for SCID GAD. After adjusting for weights, SCID GAD was estimated in 438.7 (11%, 95%CI: 7 to 16%) of GAD-2 (Yes/No) positives. Whereas, 5633 (100%) of GAD-2 (Yes/No) negatives and 3678 (89%, 95%CI: 84 to 93%) GAD-2 (Yes/No) positives did not meet criteria for SCID GAD (see online supplementary files 2 & 4 for cross-tabulation and proportions). The weighted sensitivity was 1, specificity 0.60, PPV 0.11, NPV 1, LR+ 2.53 and LR- 0.

Diagnostic accuracy of the GAD-2 screening questions in identifying any anxiety disorder

SCID anxiety disorders (including GAD, PTSD and OCD) were found in 73 (18%) of GAD-2 (<3) negatives and 57 (50%) of GAD-2 (≥3) positive women. After adjusting for weights, an estimated 1125 (13%, 95%CI: 10 to 18%) of GAD-2 (<3) negatives and 403.6 (36%, 95%CI: 23 to 51%) of GAD-2 (≥3) positives met criteria for any SCID anxiety disorder (including PTSD and OCD). 7268 (87%, 95%CI: 82 to 90%) GAD-2 (<3) negatives and 723.1 (64%, 95%CI: 49 to 77%) GAD-2 (≥3) positives had no anxiety disorders (see online supplementary file 5 & 6 for cross-tabulation and proportions). Weighted sensitivity was 0.26, specificity 0.91, PPV 0.36, NPV 0.87, LR+ 2.92 and LR- 0.81. When scoring of the GAD-2 was changed to ≥1 (Yes/No response), 22 (10%) of GAD-2 (Yes/No) negative and 108 (37%) of GAD-2 (Yes/No) positive women met criteria for any anxiety disorder (including PTSD and OCD). After adjusting for weights, 452.8 (8%, 95%CI: 5 to 13%) of GAD-2 (Yes/No) negatives and 1076 (27%, 95%CI: 20 to 35%) of GAD-2 (Yes/No) positives met criteria for any SCID anxiety disorder (including PTSD and OCD). Whereas, 5110 (92%, 95%CI: 87 to 95%) GAD-2 (Yes/No) negatives and 2881 (73%, 95%CI: 65 to 80%) GAD-2 (Yes/No) positives had no anxiety disorders (see online supplementary files 5 & 7 for cross-tabulation and proportions). Weighted sensitivity was 0.70, specificity 0.64, PPV 0.27, NPV 0.92, LR+ 1.95 and LR- 0.46. The exclusion of PTSD and OCD to the group of "any" anxiety disorders made little difference (see Table 3 and online supplementary file 8 for cross-tabulation). Sensitivity analysis of when PTSD missing data were considered as cases of PTSD and not cases of PTSD also did not make much difference (see Table 3 and online supplementary file 9 for cross-tabulations).

[INSERT TABLE 3 HERE]

For peer review only - http://bmjopen.bmj.com/site/about/guidelines.xhtml

DISCUSSION

In this inner city maternity population, the population prevalence estimated for Generalised Anxiety Disorder (GAD) was 5% (95%CI: 3 to 6%) and for all anxiety disorders was 17% (95%CI: 12 to 21%), in line with other studies.[11] The population prevalence estimated for PTSD was lower (0.8%, 95%CI: 0 to 1%) than the mean prevalence of 3.86% reported in a recent systematic review of PTSD identified by interviews during pregnancy.[27] However, in our study, when missing data were assumed to indicate cases of PTSD, the prevalence estimate was 4% (95%CI: 2 to 6%), highlighting the possibility of barriers to disclosure of trauma and associated symptoms. As others have reported,[28 29] comorbidity is also common and we found estimates for comorbid depression and GAD (2%, 95%CI: 1 to 3%) that were similar to previous estimates derived from representative samples and utilising diagnostic clinical interviews (GAD and depression 2% (95%CI: 0 to 3%).[28] However, the population prevalence estimated in our sample of comorbid depression and any anxiety (5%, 95%CI: 2 to 7%) was slightly lower than previous estimates (7%, 95%CI: 3 to 11).[28]

This study makes a novel contribution to the gap in the literature by formally examining the diagnostic accuracy of the GAD-2 screening questionnaire for women during early pregnancy. The GAD-2 (using a cut-off of ≥3; as recommended by NICE) had a reasonable likelihood ratio when used in early pregnancy for identifying generalised anxiety disorder (LR+: 7 i.e. above 5, which has been suggested to indicate a potentially useful tool in clinical practice)[30] . However, GAD is not very common and the positive predictive value is low (26%). Furthermore, the diagnostic accuracy of the GAD-2 for identifying other anxiety disorders was poor (LR+2.92). This evidence suggests that the GAD-2 is not a helpful tool for maternity services as it will generate many false positives.

There are several strengths to this study. Firstly, we recruited a stratified representative sample of women using language interpreters to facilitate inclusion of non-English speaking women (Howard et al, 2018).[21] Secondly, we used an efficient and robust sampling design which facilitated the estimation of population prevalence's and we used a gold standard diagnostic interview. Limitations include recruitment from a single maternity site in London, though the single South London maternity site utilised in this study included a very ethnically and socioeconomically diverse population. There were some missing data (though this was rare other than for PTSD), and timing of administering the GAD-2 in early pregnancy may have over-inflated the risk of "false positives".

For peer review only - http://bmjopen.bmj.com/site/about/guidelines.xhtml

Implications

This study does not support the NICE recommendation to use the GAD-2 in early pregnancy, due to its low positive predictive value even for generalised anxiety disorder (when applying a cut-off of both ≥3 or ≥1 indicating a yes response) and low effectiveness for "any anxiety disorder". Its accuracy later in pregnancy warrants further study but a recent systematic review reported the prevalence of self-reported anxiety symptoms to increase through pregnancy (trimester 1: 18%, trimester 2: 19%, trimester 3: 25%); [11] diagnostic accuracy is therefore unlikely to improve.

Following the NICE CG192 guideline recommendations, some services have already implemented the GAD-2 in routine practice. The findings from the current paper challenge the use of expert consensus and extrapolation from evidence derived from the general population to pregnant women when making NICE recommendations specifically for pregnant women. We argue that recommendations for pregnant women should be evidence based and propose that, at present, only the NICE recommendation on use of the Whooley questions is supported by evidence.[21] Currently, the evidence suggests that implementation of routine use of the GAD-2 is unwarranted. This brings us to a number of potential unexplored future research directions, which include a comprehensive diagnostic accuracy study, including data on acceptability, of the full GAD-7 questionnaire in pregnancy and the three "anxiety" items in the Edinburgh Postnatal Depression Scale in pregnancy. For the GAD-7, currently there is no evidence. There is some, conflicting evidence for the three "anxiety items" in the Edinburgh Postnatal Depression Scale, but as others have suggested, research is needed to determine their validity, reliability and diagnostic accuracy as a measure of antenatal anxiety disorders.[31 32] Furthermore, use of other routinely collected data including the Whooley questions could be used in a model (using predictive modelling techniques) to identify anxiety disorders or perhaps even more importantly, "any mental disorder". Finally, further research is needed to examine ways to overcome the barriers to disclosure of trauma and trauma-related symptoms in pregnancy. This suggests that more sensitive methods need to be developed to ask women about trauma.

Conclusions

This study suggests that anxiety disorders are common in early pregnancy but the GAD-2 screening measure is not useful during early pregnancy. In addition, even if asked directly, PTSD may be particularly difficult to detect in routine practice and further research is needed on how to overcome barriers to disclosure of traumatic symptoms.

For peer review only - http://bmjopen.bmj.com/site/about/guidelines.xhtml

Acknowledgements: We are grateful for the advice received regarding the WENDY study from our Patient and Public Advisory Group, our Programme Steering Committee (Prof Rona McCandlish (Chair), Dr Heather O'Mahen, Dr Pauline Slade, Ceri Rose and Rosemary Jones) and our Data Monitoring and Ethics Committee (Roch Cantwell (chair), Liz McDonald-Clifford, Marian Knight, Stephen Bremner). We also want to take the opportunity to thank the women who participated in this study.

Author Contributions: LH, DB, JM & AP conceived, planned and obtained funding for the ESMI programme of research and they, with KT, wrote the first draft of the WENDY study protocol. JD was involved in modifying study protocol. SN was involved in the literature search, planning and carrying out statistical analysis, interpretation of data and drafted the manuscript. ER and AP provided guidance on statistical analysis. Both LH and ER were involved with interpretation of data and revision on manuscript drafts. All authors commented on subsequent drafts, interpretation of data, and approved final version of manuscript. Professor Louise Howard (senior author and chief investigator) is the guarantor of the study.

Funding: This paper summarises independent research funded by the National Institute for Health Research (NIHR) under the Programme Grants for Applied Research programme (ESMI Programme: grant reference number RP-PG-1210-12002) and the National Institute for Health Research (NIHR) / Wellcome Trust Kings Clinical Research Facility and the NIHR Biomedical Research Centre and Dementia Unit at South London and Maudsley NHS Foundation Trust and Kings College London. The views expressed are those of the author(s) and not necessarily those of the NHS, the NIHR or the Department of Health. The study team acknowledges the study delivery support given by the South London Clinical Research Network. The funder had no role in the design or conduct of the study; the collection, management, analysis, and interpretation of the data; or the writing of the article.

Competing interests: All authors have completed the ICMJE uniform disclosure form at www.icmje.org/coi_disclosure.pdf and declare support from the National Institute for health Research for the submitted work; no financial relationships with any organisations that might have an interest in the submitted work in the previous three years; no other relationships or activities that could appear to have influenced the submitted work. Louise Howard (senior author and chief investigator), chaired the

For peer review only - http://bmjopen.bmj.com/site/about/guidelines.xhtml

NICE CG192 guidelines development group on antenatal and postnatal mental health in 2012-4. No other conflicts of interest.

Data sharing and access to study protocol: Full study protocol (approved by Research Ethics Committee) and patient level data is available from Chief Investigator Professor Louise Howard (louise.howard@kcl.ac.uk). Consent for data sharing was not obtained but the presented data are anonymised and risk of identification is very low.

For peer review only

For peer review only - http://bmjopen.bmj.com/site/about/guidelines.xhtml

FIGURE

Figure 1: Flow chart of women through the WENDY study and GAD-2 data

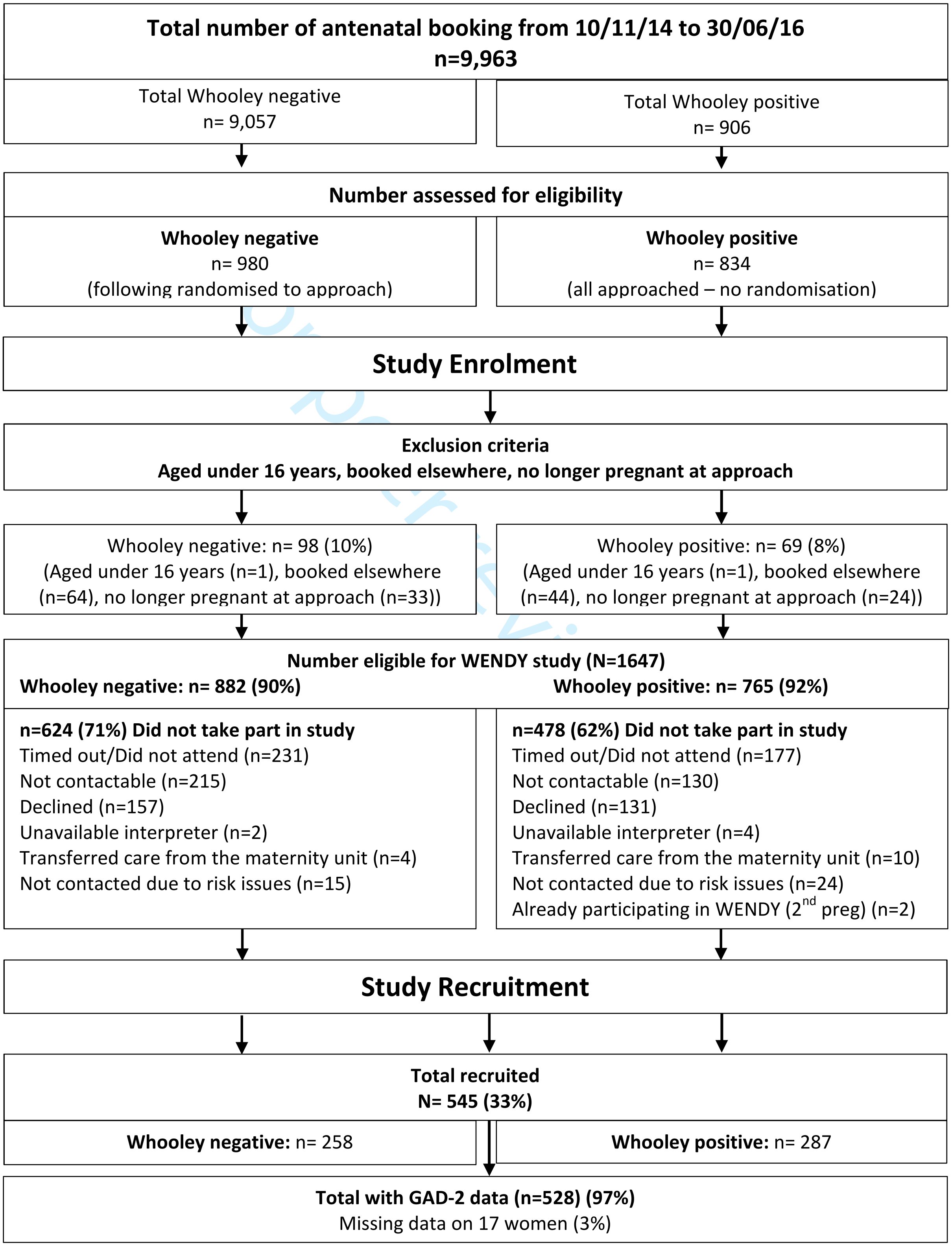

For peer review only - http://bmjopen.bmj.com/site/about/guidelines.xhtml

TABLES

Table 1: Socio-demographics and characteristics of participants with GAD-2 data included in diagnostic accuracy analyses (n=528)

	GAD-2 Negative Score < 3 N=409	GAD-2 Positive Score ≥ 3 N=119	p	Overall (total) N=528
**Age (years)**			0.126	
16 – 19	4 (1%)	3 (3%)		7 (1%)
20-29	104 (25%)	40 (34%)		144 (27%)
30-39	267 (65%)	69 (58%)		336 (64%)
40+	34 (8%)	7 (6%)		41 (8%)
**Ethnicity**			0.384	
White	223 (55%)	56 (47%)		279 (53%)
Black/ Caribbean	123 (30%)	46 (39%)		169 (32%)
Asian/Asian British	19 (5%)	3 (3%)		22 (4%)
Mixed/Multiple ethnicity	17 (4%)	5 (4%)		22 (4%)
Other	27 (7%)	9 (8%)		36 (7%)
**Highest Education level**			0.006	
None/school qualifications	38 (9%)	24 (20%)		62 (12%)
College/Diploma/Higher/Certificate/training	147 (36%)	42 (35%)		189 (36%)
Degree level/ Postgraduate qualifications	224 (55%)	53(45%)		277 (52%)
**Employment status**[1]			0.010	
Employed	271 (66%)	69 (58%)		340 (65%)
Student	19 (5%)	2 (2%)		21 (4%)
Unemployed	42 (10%)	19 (16%)		61 (12%)
Homemaker	58 (14%)	14 (12%)		72 (14%)
Not working due to illness/Other	18 (4%)	14 (12%)		32 (6%)
**Income**[2]			<0.001	
< £15000	41 (13%)	32 (35%)		73 (18%)
£15,000-£30,999	54 (17%)	16 (17%)		70 (17%)
£31,000-£45,999	50 (16%)	10 (11%)		60 (15%)
£46,000-£60,999	52 (16%)	10 (11%)		62 (15%)
£61,000 or more	119 (38%)	24 (26%)		143 (35%)
**Relationship status**			<0.001	
Single	34 (8%)	23 (19%)		57 (11%)
Partner but not cohabiting	57 (14%)	22 (18%)		79 (15%)
Married/cohabiting	313 (77%)	70 (59%)		383 (72%)
Separated/divorced	5 (1%)	4 (3%)		9 (2%)
**Multiparous**				
No	202 (49%)	62 (52%)	0.603	264 (50%)
Yes	207 (51%)	57 (48%)		264 (50%)
**Planned pregnancy**				
Yes	280 (68%)	67 (56%)	0.014	347 (66%)
**Late booker**				
Yes	344 (84%)	94 (79%)	0.191	438 (83%)
**Translator required**				
Yes	25 (6%)	13 (11%)	0.074	38 (7%)

[1] 2 participants had missing data on employment status (1 GAD-2 positive and 1 GAD-2 negative)
[2] 120 participants had missing data on income (93 GAD-2 negatives, 27 GAD-2 positives)

For peer review only - http://bmjopen.bmj.com/site/about/guidelines.xhtml

Table 2: Co-morbidities in the WENDY sample including unweighted count (%) & *weighted population prevalence estimates % (95% CI)*

**Disorder** **N**	Depression[1] (n=545)	GAD (n=544)	Panic disorder (n=545)	Agoraphobia (n=544)	Social phobia (n=545)	Specific phobia (n=544)	PTSD (n=524)	OCD (n=545)	Eating disorders[2] (n=543)
Depression[1] (n=545)	-	*2% (1 - 3%)*	*0.2% (0 - 2%)*	*0.06% (0 – 0.3%)[3]*	*1% (0 – 2%)*	*1% (0 – 3%)*	*0.4% (0 – 2%)*	*1% (0 - 2%)*	*0.2% (0-0.4%)[3]*
GAD (n=544)	39/544 (7%)	-	*0.13% (0.05 - 0.34%)[3]*	*0.35% (0 - 2%)[3]*	*1% (0 – 2%)*	*0.5% (0 - 1%)*	*0%*	*0.1% (0-0.3%)[3]*	*0.2% (0-0.4%)[3]*
Panic disorder (n=545)	5/545 (1%)	4/544 (1%)	-	*0%*	*0.03% (0-0.2%)[3]*	*0%*	*0%*	*0.03% (0-0.2%)[3]*	*0.03% (0-0.2%)[3]*
Agoraphobia (n=544)	2/544 (0.4%)	1/543 (0.2%)	0/544 (0%)	-	*0.4 (0.05-2%)[3]*	*0%*	*0%*	*0%*	*0%*
Social phobia (n=545)	9/545 (1.7%)	6/544 (1%)	1/545 (0.2%)	1/544 (0.2%)	-	*1% (0-2%)*	*0.07% (0.02-0.3%)[3]*	*0.5% (0 - 2%)*	*0.1% (0.05-0.4)[3]*
Specific phobia (n=544)	13/544 (2%)	6/543 (1%)	0/544 (0%)	0/544 (0%)	5/544 (1%)	-	*0%*	*1% (0.2 – 3%)[3]*	*0.03% (0 – 0.2%)[3]*
PTSD (n=524)	11/524 (2%)	0/523 (0%)	0/524 (0%)	0/524 (0%)	2/524 (0.4%)	0/524 (0%)	-	*0.03% (0 – 0.2%)[3]*	*0%*
OCD (n=545)	11/545 (2%)	4/544 (1%)	1/545 (0.2%)	0/544 (0%)	5/545 (1%)	4/544 (1%)	1/524 (0.2%)	-	*0.08% (0.02 – 0.3%)[3]*
Eating disorders[2] (n=543)	4/543 (1%)	4/543 (1%)	1/543 (0.2%)	0/542 (0%)	4/543 (1%)	1/542 (0.2%)	0/522 (0%)	2/543 (0.4%)	-

[1] Depression includes all types of depression (major depressive disorder, mixed anxiety and depression).
[2] Eating disorders includes anorexia nervosa (including atypical), bulimia nervosa, binge eating disorder, purging disorder and other specified or eating disorder.
[3] Bootstrap CI could not be performed due to small sample size. Weighted CI are presented which take into account sampling and missing SCID.

For peer review only - http://bmjopen.bmj.com/site/about/guidelines.xhtml

Table 3: Sensitivity, specificity, PPV, NPV, LR+ and LR- of the GAD-2 questions (n=528)

GAD-2 cut-off	GAD	Any anxiety disorders (including PTSD and OCD)			Any anxiety disorders (excluding PTSD and OCD)[2]
		19 missing data on PTSD (n=516)[1]	If missing cases considered as PTSD cases	If missing cases considered as not PTSD cases	
Cut-off ≥ 3					
Sensitivity	0.69	0.26	0.26	0.26	0.26
Specificity	0.91	0.91	0.91	0.91	0.90
PPV	0.26	0.36	0.38	0.34	0.31
NPV	0.98	0.87	0.85	0.87	0.88
LR+	7.35	2.92	2.84	2.82	2.71
LR-	0.34	0.81	0.82	0.81	0.82
Cut-off ≥ 1 (Yes/No)					
Sensitivity	1	0.70	0.70	0.70	0.68
Specificity	0.60	0.64	0.64	0.63	0.62
PPV	0.11	0.27	0.30	0.26	0.23
NPV	1	0.92	0.91	0.92	0.92
LR+	2.53	1.95	1.95	1.90	1.81
LR-	0	0.46	0.47	0.47	0.51

[1] 19 participants had missing data on the PTSD module. 7 of these participants met criteria for other anxiety disorders. Therefore, the total sample size for any anxiety disorders was 516.
[2] We excluded PTSD and OCD to present the difference, as DSM-5 no longer considered PTSD and OCD as anxiety disorders.

For peer review only - http://bmjopen.bmj.com/site/about/guidelines.xhtml

REFERENCES

1. Kessler RC, Petukhova M, Sampson NA, et al. Twelve-month and lifetime prevalence and lifetime morbid risk of anxiety and mood disorders in the United States. *International journal of methods in psychiatric research* 2012;21(3):169-84.
2. Li SH, Graham BM. Why are women so vulnerable to anxiety, trauma-related and stress-related disorders? The potential role of sex hormones. *The Lancet Psychiatry* 2017;4(1):73-82.
3. Goodman JH, Chenausky KL, Freeman MP. Anxiety disorders during pregnancy: a systematic review. *The Journal of clinical psychiatry* 2014;75(10):1153-84.
4. Martini J, Petzoldt J, Einsle F, et al. Risk factors and course patterns of anxiety and depressive disorders during pregnancy and after delivery: A prospective-longitudinal study. *Journal of affective disorders* 2015;175:385-95.
5. Stein A, Pearson RM, Goodman SH, et al. Effects of perinatal mental disorders on the fetus and child. *The Lancet* 2014;384(9956):1800-19.
6. Glover V. Maternal depression, anxiety and stress during pregnancy and child outcome; what needs to be done. *Best practice & research Clinical obstetrics & gynaecology* 2014;28(1):25-35.
7. Newman L, Judd F, Komiti A. Developmental implications of maternal antenatal anxiety mechanisms and approaches to intervention. *Translational Developmental Psychiatry* 2017;5(1):1309879.
8. Barker ED, Jaffee SR, Uher R, et al. The contribution of prenatal and postnatal maternal anxiety and depression to child maladjustment. *Depress Anxiety* 2011;28(8):696-702. doi: 10.1002/da.20856 [published Online First: 2011/07/20]
9. Blair MM, Glynn LM, Sandman CA, et al. Prenatal maternal anxiety and early childhood temperament. *Stress* 2011;14(6):644-51. doi: 10.3109/10253890.2011.594121 [published Online First: 2011/07/28]
10. O'Connor TG, Heron J, Golding J, et al. Maternal antenatal anxiety and children's behavioural/emotional problems at 4 years. Report from the Avon Longitudinal Study of Parents and Children. *Br J Psychiatry* 2002;180:502-8.
11. Dennis C-L, Falah-Hassani K, Shiri R. Prevalence of antenatal and postnatal anxiety: systematic review and meta-analysis. *Br J Psychiatry* 2017;210(5):315-23. doi: 10.1192/bjp.bp.116.187179
12. National Institute for Health and Care Excellence. Antenatal and Postnatal Mental Health guidelines - Clinical guidelines CG192. London: National Institute for Health and Care Excellence (NICE), 2014.
13. Howard LM, Megnin-Viggars O, Symington I, et al. Antenatal and postnatal mental health: summary of updated NICE guidance. *BMJ* 2014;349:g7394.
14. Fontein-Kuipers Y, Nieuwenhuijze M, Ausems M, et al. Antenatal interventions to reduce maternal distress: a systematic review and meta-analysis of randomised trials. *BJOG: An International Journal of Obstetrics & Gynaecology* 2014;121(4):389-97.
15. Kelly RH, Danielson BH, Zatzick DF, et al. Chart-recorded psychiatric diagnoses in women giving birth in California in 1992. *Am J Psychiatry* 1999;156(6):955-57.
16. Darwin Z, McGowan L, Edozien LC. Antenatal mental health referrals: Review of local clinical practice and pregnant women ʼs experiences in England. *Midwifery* 2015;31(3):e17-e22.
17. Evans K, Spiby H, Morrell CJ. A psychometric systematic review of self-report instruments to identify anxiety in pregnancy. *Journal of advanced nursing* 2015;71(9):1986-2001.
18. Plummer F, Manea L, Trepel D, et al. Screening for anxiety disorders with the GAD-7 and GAD-2: a systematic review and diagnostic metaanalysis. *General Hospital Psychiatry* 2016;39:24-31.

For peer review only - http://bmjopen.bmj.com/site/about/guidelines.xhtml

19. First MB, Spitzer RL, Gibbon M, et al. Structured clinical interview for DSM-IV-TR axis I disorders, research version, patient edition. *New York: Biometrics Research, New York State Psychiatric Institute* 2002
20. Diagnostic A. statistical manual (DSM-V). *Washington, DC: American Psychiatric Association* 2013
21. Howard LM, Ryan EG, Trevillion K, et al. Accuracy of the Whooley questions and the Edinburgh Postnatal Depression Scale in identifying depression and other mental disorders in early pregnancy. *The British Journal of Psychiatry* 2018;212(1):50-56.
22. Spitzer RL, Kroenke K, Williams JB, et al. A brief measure for assessing generalized anxiety disorder: the GAD-7. *Archives of internal medicine* 2006;166(10):1092-97.
23. Kroenke K, Spitzer RL, Williams JBW, et al. Anxiety disorders in primary care: prevalence, impairment, comorbidity, and detection. *Ann Intern Med* 2007;146(5):317-25.
24. Celano CM, Suarez L, Mastromauro C, et al. Feasibility and utility of screening for depression and anxiety disorders in patients with cardiovascular disease. *Circulation: Cardiovascular Quality and Outcomes* 2013;6(4):498-504.
25. StataCorp. Stata Statistical Software: Release 15. College Station, TX: StataCorp LLC., 2017.
26. Howard L, Ryan E, Trevillion K, et al. The accuracy of the Whooley questions and the Edinburgh Postnatal Depression Scale in identifying depression and other mental disorders in early pregnancy. *The British Journal of Psychiatry* In Press
27. Yildiz P, Ayers S, Phillips L. The prevalence of posttraumatic stress disorder in pregnancy and after birth: A systematic review and meta-analysis. *Journal of Affect Disorders* 2017;208:634-45. doi: 10.1016/j.jad.2016.10.009
28. Falah-Hassani K, Shiri R, Dennis C-L. The prevalence of antenatal and postnatal co-morbid anxiety and depression: a meta-analysis. *Psychological Medicine* 2017:1-13.
29. Dindo L, Elmore A, O'Hara M, et al. The comorbidity of Axis I disorders in depressed pregnant women. *Archives of Women's Mental Health* 2017:1-8.
30. Grimes DA, Schulz KF. Refining clinical diagnosis with likelihood ratios. *The Lancet* 2005;365(9469):1500-05.
31. Matthey S, Fisher J, Rowe H. Using the Edinburgh postnatal depression scale to screen for anxiety disorders: Conceptual and methodological considerations. *Journal of Affective Disorders* 2013;146:224-30.
32. Milgrom J, Gemmill AW. Screening for perinatal depression. *Best Practice & Research Clinical Obstetrics & Gynaecology* 2014;28(1):13-23.

For peer review only

For peer review only - http://bmjopen.bmj.com/site/about/guidelines.xhtml

Online supplementary file 1

Table showing characteristics of wider base population and study population

	Base population*	WENDY study sample N=545	Sample with GAD-2 data N=528
**Age (years)**	Mean: 31.67<br>Range: 14 – 52	Mean: 32.85<br>range: 16 – 47.5	Mean: 32.84<br>Range: 16 – 47.5
16 – 19	232 (2%)	8 (1%)	7 (1%)
20 – 29	3048 (30%)	150 (28%)	144 (27%)
30 – 39	6240 (61%)	341 (63%)	336 (64%)
40+	705 (7%)	46 (8%)	41 (8%)
**Ethnicity**			
White	4914 (51%)	284 (52%)	279 (53%)
Black/ Caribbean	3162 (33%)	177 (32%)	169 (32%)
Asian/Asian British	594 (6%)	25 (5%)	22 (4%)
Mixed/Multiple ethnicity	308 (3%)	23 (4%)	22 (4%)
Other	646 (7%)	36 (7%)	36 (7%)
**Other children**			
None	5077 (50%)	271 (50%)	264 (50%)
1	3209 (31%)	175 (32%)	167 (32%)
2 or more	1939 (19%)	99 (18%)	97 (18%)

*Base population covers women seen from 1.11.2014 - 30.6.2016, missing data not included.

For peer review only

Online supplementary file 2

Table of weighted population prevalence for calculations of sensitivity and specificity for GAD including count and proportion % (95% CI)

	**GAD-2 (<3) negative**	**GAD-2 (≥3) positive**
**No GAD**	8439<br>98% (97 – 99%)	872.2<br>74% (60 – 85%)
**SCID GAD**	136.5<br>2% (1 – 3%)	302.2<br>26% (15 – 40%)
	**GAD-2 (Yes/No) negative**	**GAD-2 (Yes/No) positive**
**No GAD**	5633<br>100%	3678<br>89% (84 – 93%)
**SCID GAD**	0<br>0%	438.7<br>11% (7 – 16%)

For peer review only

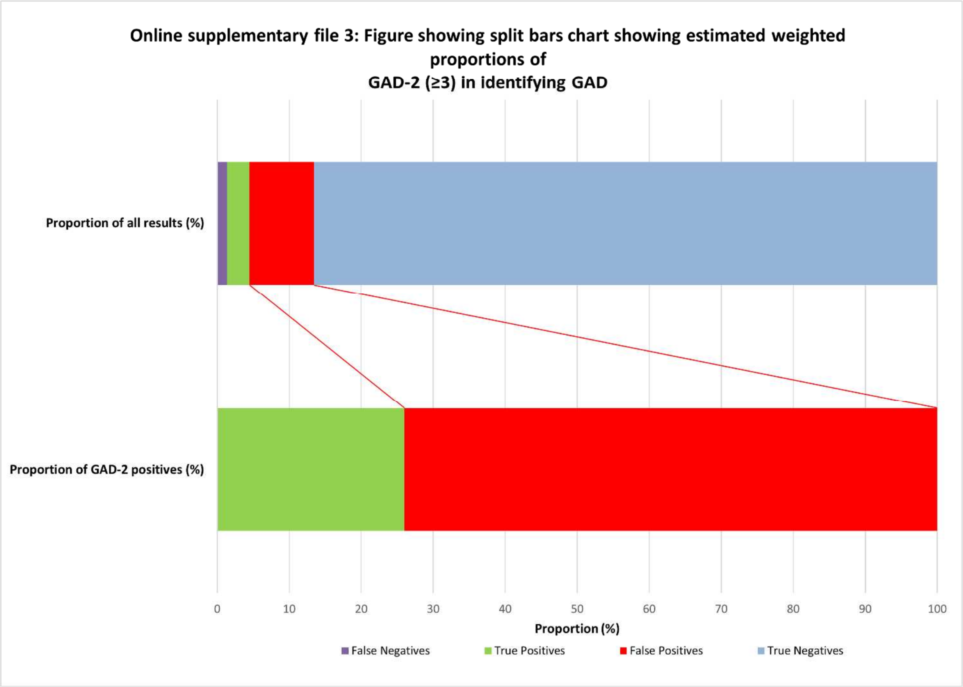

254x190mm (300 x 300 DPI)

For peer review only - http://bmjopen.bmj.com/site/about/guidelines.xhtml

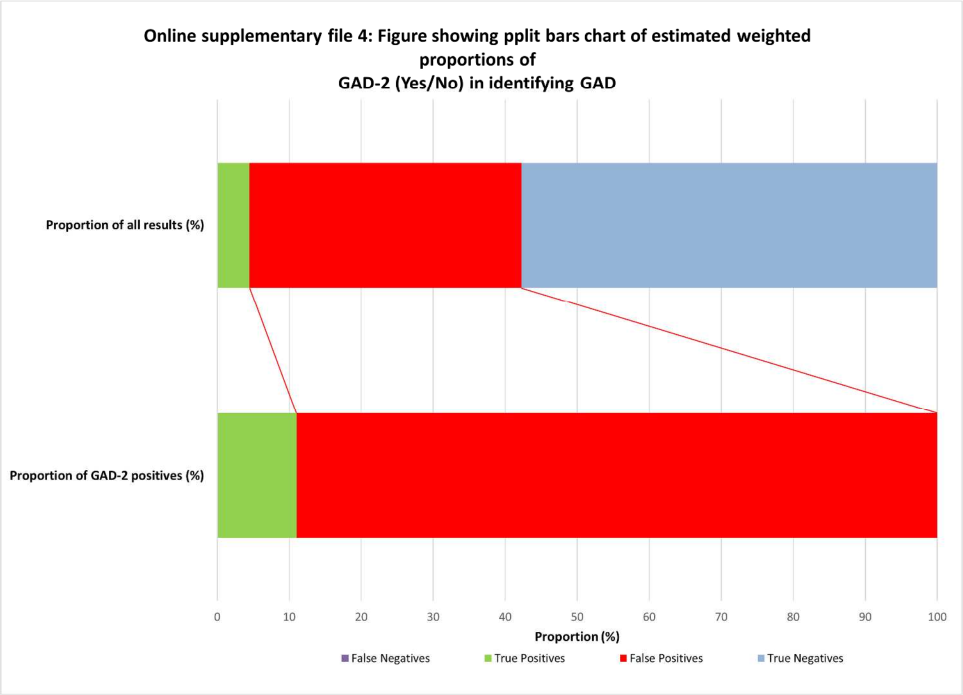

254x190mm (300 x 300 DPI)

Online supplementary file 5

Table of weighted population prevalence for calculations of sensitivity and specificity for any anxiety disorder (including PTSD and OCD) including count and proportion % (95% CI)

	GAD-2 (<3) negative	GAD-2 (≥3) positive
**No anxiety**	7268<br>87% (82 – 90%)	723.1<br>64% (49 – 77%)
**SCID any anxiety**	1125<br>13% (10 – 18%)	403.6<br>36% (23 – 51%)
	**GAD-2 (Yes/No) negative**	**GAD-2 (Yes/No) positive**
**No anxiety**	5110<br>92% (87 – 95%)	2881<br>73% (65 – 80%)
**SCID any anxiety**	452.8<br>8% (5 – 13%)	1076<br>27% (20 – 35%)

For peer review only

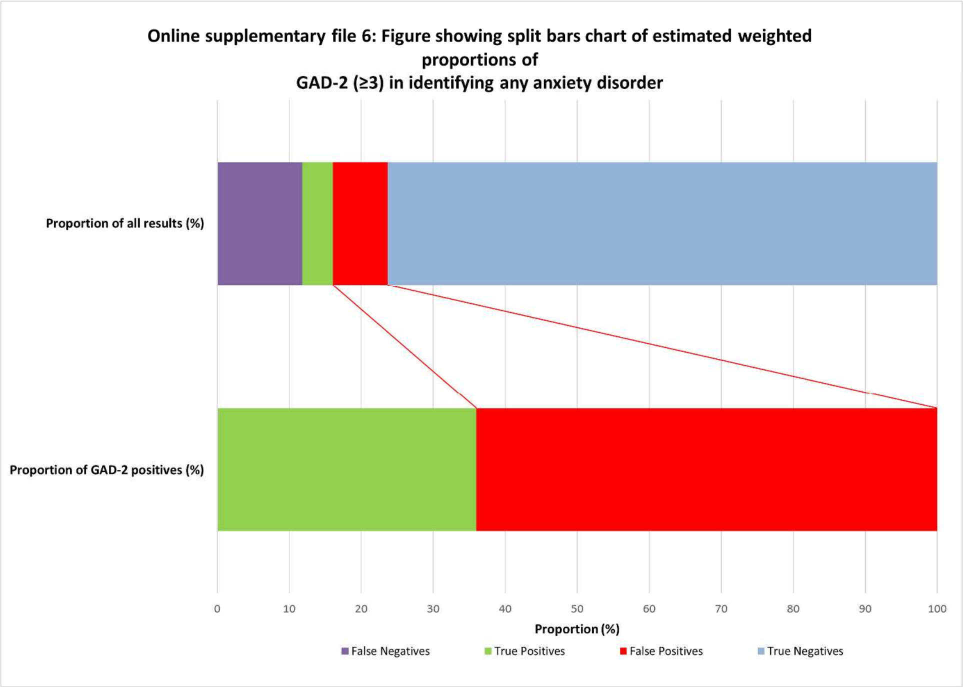

254x190mm (300 x 300 DPI)

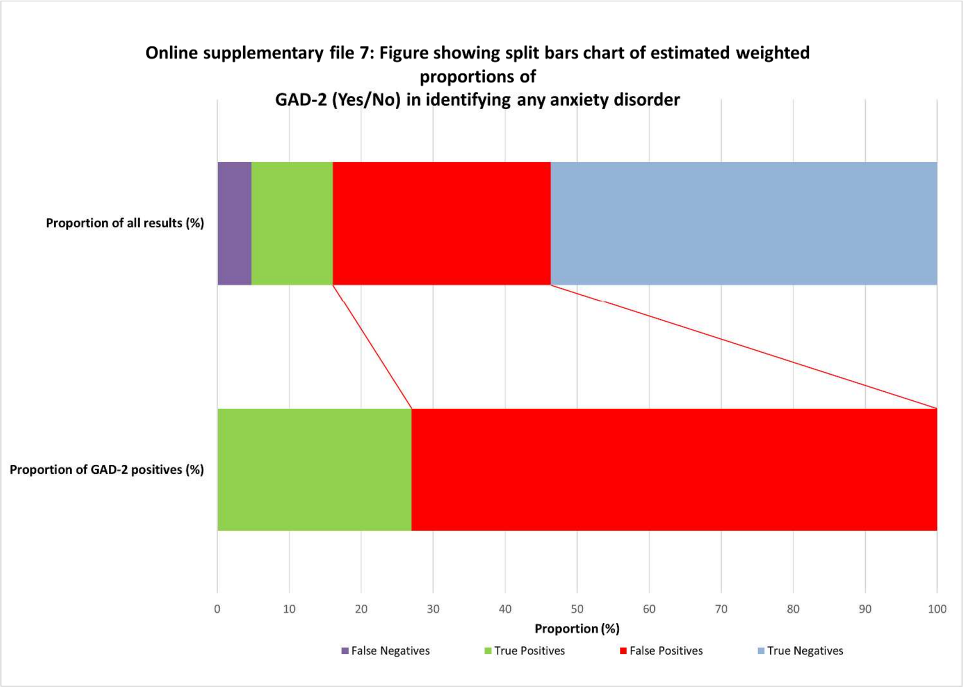

254x190mm (300 x 300 DPI)

Online supplementary file 8

Table of weighted population prevalence's for calculations of sensitivity and specificity for any anxiety disorder (excluding PTSD and OCD)

	**GAD-2 (<3) negative**	**GAD-2 (≥3) positive**
**No anxiety**	7536<br>88% (83 – 91%)	805.5<br>69 (54 – 80%)
**SCID any anxiety**	1039<br>12% (9 – 17%)	368.8<br>31 (20 – 46%)
	**GAD-2 (Yes/No) negative**	**GAD-2 (Yes/No) positive**
**No anxiety**	5187<br>92% (87 – 95%)	3155<br>77% (69 – 83%)
**SCID any anxiety**	446.5<br>8% (5 – 13%)	961.3<br>23% (17 – 31%)

For peer review only

Online supplementary file 9

Table of weighted population prevalence's for calculations of sensitivity and specificity for any anxiety disorder (including PTSD and OCD) (missing PTSD data considered as PTSD cases)

	**GAD-2 (<3) negative**	**GAD-2 (≥3) positive**
**No anxiety**	7268<br>85% (80 – 89%)	723.1<br>62% (47 – 74%)
**SCID any anxiety**	1307<br>15% (11 – 20%)	451.3<br>38% (26 – 53%)
	**GAD-2 (Yes/No) negative**	**GAD-2 (Yes/No) positive**
**No anxiety**	5110<br>91% (85 – 94%)	2881<br>70% (62 – 77%)
**SCID any anxiety**	523<br>9% (6 – 15%)	1235<br>30% (23 – 38%)

Table of weighted population prevalence's for calculations of sensitivity and specificity for any anxiety disorder (including PTSD and OCD) (missing PTSD data considered as not PTSD cases)

	**GAD-2 (<3) negative**	**GAD-2 (≥3) positive**
**No anxiety**	7450<br>87% (82 – 90%)	770.8<br>66% (51 – 78%)
**SCID any anxiety**	1125<br>13% (10 – 18%)	403.6<br>34% (22 – 49%)
	**GAD-2 (Yes/No) negative**	**GAD-2 (Yes/No) positive**
**No anxiety**	5181<br>92%(87 – 95%)	3040<br>74% (66 – 80%)
**SCID any anxiety**	452.8<br>8% (5 – 13%)	1076<br>26 (20 – 34%)

For peer review only - http://bmjopen.bmj.com/site/about/guidelines.xhtml

Section & Topic	No	Item	Reported on page #
**TITLE OR ABSTRACT**			
	**1**	Identification as a study of diagnostic accuracy using at least one measure of accuracy (such as sensitivity, specificity, predictive values, or AUC)	2
**ABSTRACT**			
	**2**	Structured summary of study design, methods, results, and conclusions (for specific guidance, see STARD for Abstracts)	2
**INTRODUCTION**			
	**3**	Scientific and clinical background, including the intended use and clinical role of the index test	4
	**4**	Study objectives and hypotheses	5
**METHODS**			
Study design	**5**	Whether data collection was planned before the index test and reference standard were performed (prospective study) or after (retrospective study)	6
Participants	**6**	Eligibility criteria	6 & figure 1
	**7**	On what basis potentially eligible participants were identified (such as symptoms, results from previous tests, inclusion in registry)	5, 6 & figure 1
	**8**	Where and when potentially eligible participants were identified (setting, location and dates)	5, 6 & figure 1
	**9**	Whether participants formed a consecutive, random or convenience series	5, 6 & figure 1
Test methods	**10a**	Index test, in sufficient detail to allow replication	6
	**10b**	Reference standard, in sufficient detail to allow replication	7
	**11**	Rationale for choosing the reference standard (if alternatives exist)	7
	**12a**	Definition of and rationale for test positivity cut-offs or result categories of the index test, distinguishing pre-specified from exploratory	6
	**12b**	Definition of and rationale for test positivity cut-offs or result categories of the reference standard, distinguishing pre-specified from exploratory	7
	**13a**	Whether clinical information and reference standard results were available to the performers/readers of the index test	6
	**13b**	Whether clinical information and index test results were available to the assessors of the reference standard	7
Analysis	**14**	Methods for estimating or comparing measures of diagnostic accuracy	8
	**15**	How indeterminate index test or reference standard results were handled	N/A
	**16**	How missing data on the index test and reference standard were handled	8
	**17**	Any analyses of variability in diagnostic accuracy, distinguishing pre-specified from exploratory	8
	**18**	Intended sample size and how it was determined	6 & Figure 1
**RESULTS**			
Participants	**19**	Flow of participants, using a diagram	Figure 1
	**20**	Baseline demographic and clinical characteristics of participants	9 & Table 1
	**21a**	Distribution of severity of disease in those with the target condition	10
	**21b**	Distribution of alternative diagnoses in those without the target condition	10
	**22**	Time interval and any clinical interventions between index test and reference standard	6
Test results	**23**	Cross tabulation of the index test results (or their distribution) by the results of the reference standard	10 & 11 (online supplementary files 2, 5, 8 and 9)
	**24**	Estimates of diagnostic accuracy and their precision (such as 95% confidence intervals)	10 & 11
	**25**	Any adverse events from performing the index test or the reference standard	9
**DISCUSSION**			
	**26**	Study limitations, including sources of potential bias, statistical uncertainty, and generalisability	12
	**27**	Implications for practice, including the intended use and clinical role of the index test	13
**OTHER INFORMATION**			
	**28**	Registration number and name of registry	2
	**29**	Where the full study protocol can be accessed	15
	**30**	Sources of funding and other support; role of funders	14

For peer review only

STARD 2015

AIM

STARD stands for "Standards for Reporting Diagnostic accuracy studies". This list of items was developed to contribute to the completeness and transparency of reporting of diagnostic accuracy studies. Authors can use the list to write informative study reports. Editors and peer-reviewers can use it to evaluate whether the information has been included in manuscripts submitted for publication.

EXPLANATION

A **diagnostic accuracy study** evaluates the ability of one or more medical tests to correctly classify study participants as having a **target condition.** This can be a disease, a disease stage, response or benefit from therapy, or an event or condition in the future. A medical test can be an imaging procedure, a laboratory test, elements from history and physical examination, a combination of these, or any other method for collecting information about the current health status of a patient.

The test whose accuracy is evaluated is called **index test.** A study can evaluate the accuracy of one or more index tests. Evaluating the ability of a medical test to correctly classify patients is typically done by comparing the distribution of the index test results with those of the **reference standard**. The reference standard is the best available method for establishing the presence or absence of the target condition. An accuracy study can rely on one or more reference standards.

If test results are categorized as either positive or negative, the cross tabulation of the index test results against those of the reference standard can be used to estimate the **sensitivity** of the index test (the proportion of participants *with* the target condition who have a positive index test), and its **specificity** (the proportion *without* the target condition who have a negative index test). From this cross tabulation (sometimes referred to as the contingency or "2x2" table), several other accuracy statistics can be estimated, such as the positive and negative **predictive values** of the test. Confidence intervals around estimates of accuracy can then be calculated to quantify the statistical **precision** of the measurements.

If the index test results can take more than two values, categorization of test results as positive or negative requires a **test positivity cut-off**. When multiple such cut-offs can be defined, authors can report a receiver operating characteristic (ROC) curve which graphically represents the combination of sensitivity and specificity for each possible test positivity cut-off. The **area under the ROC curve** informs in a single numerical value about the overall diagnostic accuracy of the index test.

The **intended use** of a medical test can be diagnosis, screening, staging, monitoring, surveillance, prediction or prognosis. The **clinical role** of a test explains its position relative to existing tests in the clinical pathway. A replacement test, for example, replaces an existing test. A triage test is used before an existing test; an add-on test is used after an existing test.

Besides diagnostic accuracy, several other outcomes and statistics may be relevant in the evaluation of medical tests. Medical tests can also be used to classify patients for purposes other than diagnosis, such as staging or prognosis. The STARD list was not explicitly developed for these other outcomes, statistics, and study types, although most STARD items would still apply.

DEVELOPMENT

This STARD list was released in 2015. The 30 items were identified by an international expert group of methodologists, researchers, and editors. The guiding principle in the development of STARD was to select items that, when reported, would help readers to judge the potential for bias in the study, to appraise the applicability of the study findings and the validity of conclusions and recommendations. The list represents an update of the first version, which was published in 2003.

More information can be found on http://www.equator-network.org/reporting-guidelines/stard.

For peer review only

BMJ Open

The prevalence and identification of anxiety disorders in pregnancy: the diagnostic accuracy of the two item Generalized Anxiety Disorder scale (GAD-2)

Journal:	*BMJ Open*
Manuscript ID	bmjopen-2018-023766.R1
Article Type:	Research
Date Submitted by the Author:	10-Jul-2018

Complete List of Authors:	Nath, Selina; Institute of Psychiatry, Psychology & Neuroscience, King's College London, Health Service and Population Research Department<br>Ryan, Elizabeth ; King's College London, Biostatistics Department<br>Trevillion, Kylee; Kings College London, Health Service and Population Research Department<br>Bick, Debra; Kings College London, Florence Nightingale School of Nursing and Midwifery<br>Demilew, Jill; King's Health Partners, Women's Health Academic Centre<br>Milgrom, Jeannette; University of Melbourne Melbourne School of Psychological Sciences, School of Psychological Sciences<br>Pickles, Andrew; Kings College London<br>Howard, Louise; institute of psychiatry, health service and population research

**Primary Subject Heading**:	Diagnostics
Secondary Subject Heading:	Evidence based practice, Health services research, Mental health
Keywords:	Maternal, Anxiety disorders < PSYCHIATRY, GAD, GAD-2, Diagnostic accuracy

SCHOLARONE™
Manuscripts

Title: The prevalence and identification of anxiety disorders in pregnancy: the diagnostic accuracy of the two item Generalized Anxiety Disorder scale (GAD-2)

Corresponding Author:

Selina Nath, selina.nath@kcl.ac.uk

Section of Women's Mental Health, Health Service and Population Research Department, Institute of Psychiatry, Psychology & Neuroscience, King's College London, De Crespigny Park, London SE5 8AF, UK.

Telephone number: +44(0)20 7848 5009

Authors:

Dr Selina Nath [1] (Postdoctoral Researcher)

Dr Elizabeth G Ryan [2,7] (Clinical Trials Statistician)

Dr Kylee Trevillion [1] (Programme manager)

Prof Debra Bick [3] (Professor of Evidence Based Midwifery)

Jill Demilew [4] (Consultant Midwife at King's College Hospital)

Prof Jeannette Milgrom [5] (Professor of Psychology)

Prof Andrew Pickles [2] (Professor of Biostatistics and Psychological Methods)

Prof Louise M Howard [1,3,6] (Professor of Women's Mental Health)

[1] Section of Women's Mental Health, Health Service and Population Research Department, Institute of Psychiatry, Psychology & Neuroscience, King's College London, De Crespigny Park, London SE5 8AF, UK.

[2] Biostatistics and Health Informatics Department, Institute of Psychiatry, Psychology and Neuroscience, King's College London, De Crespigny Park, London SE5 8AF, UK.

[3] Department of Women and Children's Health, School of Life Course Sciences, Faculty of Life Sciences and Medicine, King's College London, Westminster Bridge Road, London, SE1 7EH, UK.

[4] Women's Health, King's College Hospital NHS Foundation Trust, Denmark Hill, London SE5 8RS

[5] Parent-Infant Research Institute (PIRI®), Austin Health, Melbourne School of Psychological Sciences, University of Melbourne, VIC 3010, Australia.

[6] South London and Maudsley NHS Foundation Trust, London SW2 1NU, UK.

[7] Warwick Clinical Trials Unit, Warwick Medical School, University of Warwick, Coventry, CV4 7AL, UK.

Word count: 3,883 words

For peer review only - http://bmjopen.bmj.com/site/about/guidelines.xhtml

For peer review only

ABSTRACT

Objective: To estimate the population prevalence of anxiety disorders during pregnancy and investigate the diagnostic accuracy of the GAD-2 for a) Generalised Anxiety Disorder and b) any anxiety disorder.

Design: Cross-sectional survey using a stratified sampling design. Sampling weights were used in the analysis to adjust for the bias introduced by the stratified sampling.

Setting: Inner-city maternity service, South London.

Participants: 545 pregnant women were interviewed after their first antenatal appointment; 528 provided answers on the GAD-2 questions.

Main outcome measures: Diagnosis generated by the Structured Clinical Interview for DSM-IV (SCID).

Results: Population prevalence of anxiety disorders was 17% (95%CI: 12 to 21%): 5% (95%CI: 3 to 6%) for Generalised Anxiety Disorder, 4% (95%CI: 2 to 6%) social phobia, 8% (95%CI: 5 to 11%) specific phobia and 2% (95%CI:1 to 4%) for obsessive-compulsive disorder. Post-Traumatic Stress Disorder prevalence was unclear due to higher levels of reluctance to respond to PTSD interview questions but sensitivity analyses suggest population prevalence maybe up to 4% (95%CI: 2 to 6%). Weighted sensitivity of GAD-2 for Generalised Anxiety Disorder (cut-off ≥3) was 69%, specificity 91%, positive predictive value 26%, negative predictive value 98% and Likelihood Ratio 7.35. For any anxiety disorder the weighted sensitivity was 26%, specificity 91%, positive predictive value 36%, negative predictive value 87%, and Likelihood Ratio 2.92.

Conclusions: Anxiety disorders are common but the GAD-2 generates many false positives and may therefore be unhelpful in maternity services.

Key words: Anxiety, pregnancy, prevalence, GAD-2, diagnostic accuracy

Registration: UKCRN ID: 16403 (Previously at http://public.ukcrn.org.uk/search/StudyDetail.aspx?StudyID=16403; now at https:/cpms.nihr.ac.uk).

For peer review only - http://bmjopen.bmj.com/site/about/guidelines.xhtml

ARTICLE SUMMARY:

STRENGTHS AND LIMITATIONS OF THIS STUDY:

- This study investigates the effectiveness of the Generalized Anxiety Disorder scale (GAD-2) in identifying antenatal anxiety disorders – a method suggested by NICE (CG192 2014) on the basis of expert consensus rather than research evidence.

- We recruited a representative sample of women using an efficient and robust sampling design which facilitated the estimation of population prevalence's.

- We used a gold standard diagnostic interview.

- This research was limited to recruitment from a single maternity site in London, though the single South London maternity site utilised was a very ethnically and socioeconomically diverse population.

For peer review only

For peer review only - http://bmjopen.bmj.com/site/about/guidelines.xhtml

INTRODUCTION

Anxiety disorders are more common in women than men,[1 2] and the perinatal period (i.e. pregnancy and the year after birth) has been reported as a particularly vulnerable time for the onset or relapse of anxiety disorders in women.[3 4] Antenatal anxiety disorders have been associated with adverse pregnancy outcomes, including preterm birth, low birth weight, lower Apgar scores, postpartum anxiety and depression, and adverse child developmental outcomes[5-7] including difficult temperament, increased sleep problems, bonding/attachment problems, and poorer emotional, behavioural and cognitive development.[5-10] A recent systematic review and meta-analysis reported a pooled prevalence of 4% (95%CI: 2 to 6%; 10 studies with pooled n=6910) for Generalised Anxiety Disorder (GAD) and 15% (95%CI: 9 to 21%; 9 studies with pooled n=4648) for anxiety disorders during pregnancy based on studies conducted outside of the UK where diagnostic clinical interviews were utilised, and 23% when using cut-offs on validated self-report questionnaires.[11] Anxiety disorders are treatable, so early detection and treatment during the antenatal period, when women are in regular contact with healthcare professionals could prevent adverse outcomes.[12-14] However, despite regular contact with healthcare professionals during pregnancy, antenatal mental disorders are often undetected and untreated.[15 16]

NICE (CG192; 2014) suggested that maternity professionals could consider the use of the 2-item Generalized Anxiety Disorders-2 tool (GAD-2) to identify anxiety disorders during pregnancy and after birth, though also highlighted the lack of evidence on the use of the GAD-2 in early pregnancy. The recommendation was therefore driven by concern about the high prevalence of anxiety disorders (NICE 2014).[12 17] This extends the focus of early intervention from a previous emphasis on identification of perinatal depression to other perinatal mental disorders, including comorbid conditions. Outside of the perinatal period, a systematic review and meta-analysis[18] of studies in men and women reported that the GAD-2 (using a cut-off of ≥3) showed fairly high pooled sensitivity 0.76 (95%CI: 0.55 to 0.89), and pooled specificity 0.81 (95%CI: 0.60 to 0.92) for GAD (5 studies with pooled n=1987). For detecting any anxiety disorder, the systematic review[18] reported a moderate sensitivity (range: 0.65 to 0.72) and unclear specificity (range: 0.39 to 0.92) (3 studies, N=1225). The diagnostic accuracy of the GAD-2 questions in identifying anxiety disorders during early pregnancy remains unstudied. As women in early pregnancy are likely to have many anxieties (e.g. over the viability of the pregnancy, decisional conflict over unplanned pregnancies), there may be high rates of “false positives” when using the GAD-2 in pregnancy which could result in inappropriate referrals to mental health services.

For peer review only - http://bmjopen.bmj.com/site/about/guidelines.xhtml

We therefore aimed to investigate:

1) The UK prevalence of Generalised Anxiety Disorder (GAD), other anxiety disorders (including panic disorder, agoraphobia without panic disorder, social phobia, specific phobia, Obsessive-Compulsive Disorder (OCD), and Post-Traumatic Stress Disorder (PTSD)) and comorbidity with other mental disorders during early pregnancy.
2) The sensitivity, specificity, Positive Predictive Value (PPV), Negative Predictive Value (NPV), positive likelihood ratio (LR+) and negative likelihood ratio (LR-) of the GAD-2 screening questions (on a Likert scale) compared with a gold standard diagnostic interview (DSM-IV)[19] for identifying GAD, and for identifying any anxiety disorders (including panic disorder, agoraphobia without panic disorder, social phobia, specific phobia, Obsessive-Compulsive Disorder (OCD), Post-Traumatic Stress Disorder (PTSD) and GAD) during early pregnancy. As DSM-V [20] no longer categorises OCD or PTSD as anxiety disorders, we also investigated diagnostic accuracy for any anxiety disorders by excluding OCD and PTSD.
3) How the sensitivity, specificity, PPV, NPV, LR+ and LR- change when the GAD-2 is scored and categorised as a Yes (cut-off 1 or more) verses No (score of 0) response instead of the conventional cut-off of 3 or more (which could be asked by midwives at the same time as the two depression screening questions).

METHODS

Study design and participants

The WEll-being in pregNancy stuDY (WENDY study) was a cross-sectional survey that recruited women from an inner city maternity service in South-east London using a sampling design stratified according to answering positive or negative (saying yes or no respectively) on either of the two Whooley questions which are routinely asked by midwives as a mental health screen during the first antenatal booking appointment ("During the past month have you often been bothered by feeling down, depressed, or hopeless?"; "During the past month have you often been bothered by having little interest or pleasure in doing things?"). A random sample of Whooley negative and all Whooley positive women were invited to participate.

For peer review only - http://bmjopen.bmj.com/site/about/guidelines.xhtml

Exclusion criteria were women under 16 years of age, women who declined to answer Whooley questions, those who had a termination or miscarriage prior to baseline interview or had already attended for their maternity booking appointment elsewhere in the UK. Eligible pregnant women who agreed to participate were recruited into the study as soon as possible after their first antenatal booking appointment, within a maximum of three weeks from the original booking appointment. Data collection of the index test (GAD-2 measure) and reference test (the gold standard diagnostic interview) were performed during the research interview, after written informed consent was obtained. Language interpreters were used where needed. For further details and power calculation of the WENDY study see Howard et al. (2018).[21] Sample size of the current analysis was determined by the number of women with available data on the index test (GAD-2). Figure 1 shows flow chart of women through the WENDY study and those used for the current analysis.

[INSERT FIGURE 1 HERE]

Research Measures

Generalized Anxiety Disorder Scale (GAD-2): The GAD-2 is a sub-scale of the Generalized Anxiety Disorder Scale GAD-7 measure[22] and is a two-item self-report screen completed by women during the research interview. The questions include "Over the last two weeks, how often have you been bothered by any of the following problems? 1) Feeling nervous, anxious or on edge; 2) Not being able to stop or control worrying"). Answers are given on a Likert scale (not at all=0, several days=1, more than half the days=2 and nearly everyday=3). Scores range from 0 - 6, with a cut-off score of 3 or more indicative of anxiety symptoms.[23] The GAD-2 has also been used where YES to either question categorises an individual as a positive screen (used in clinical practice),[24] thus a score of 1 or more would indicate a possible YES answer. In this study, women's responses to the GAD-2 questions were categorised into the following groups:

1) GAD-2 (≥3): GAD-2 positive if they scored 3 or more (conventional scoring method), GAD-2 negatives if they scored less than 3.
2) GAD-2 (Yes/No): GAD-2 positive if they scored 1 or more (indicating a "yes" to either question) which is used more in clinical practice, GAD-2 negative if scoring 0 (indicating "no" to both questions).

For peer review only - http://bmjopen.bmj.com/site/about/guidelines.xhtml

Anxiety disorders and comorbid disorders

The Structured Clinical Interview for DSM-IV Axis I Disorders (SCID-I): the SCID is a researcher-administered, semi-structured, and gold standard diagnostic interview consisting of standardised questions that correspond to each DSM-IV Axis I criteria.[19] We used the mood and anxiety disorders modules to generate diagnosis of depression, generalised anxiety disorder, panic disorder, agoraphobia without panic disorder, social phobia, specific phobia, obsessive-compulsive disorder, and post-traumatic stress disorder. Consensus on diagnosis was achieved during researcher's weekly meetings with LH. Although clinical information and GAD-2 data were available at the time of these meetings, agreement on diagnosis was reached using responses given by women during the SCID interviews (i.e. the GAD-2 items were not used to determine diagnosis of anxiety disorders).

As DSM-V[20] does not categorise OCD or PTSD as anxiety disorders and as the research version of the SCID for DSM-V was released after the start of the current study, we also carried out analyses for any anxiety disorders according to those included in DSM-V as anxiety disorders, i.e. excluding OCD and PTSD. We also used the eating disorder module to generate diagnosis for eating disorders including anorexia nervosa (including atypical), bulimia nervosa, binge eating disorder, purging disorder and other eating disorder.

Patient involvement

The development of the WENDY study, outcome measures, grant application, and study protocol were informed by our patient and carer advisory group. Meetings were held every few months to discuss the WENDY study and other related studies within a programme of work funded by NIHR (https://www.kcl.ac.uk/ioppn/depts/hspr/research/CEPH/wmh/projects/A-Z/esmi.aspx). The patient advisory group includes women with a range of mental disorders and have interest in our study programme. We circulated the results and draft manuscript (see acknowledgements) to members of the group for comments. Patients were not involved in the recruitment to and conduct of the study.

Statistical analysis

Data were managed and analysed using Stata v.15.[25] Sampling weights were used to adjust for the oversampling of Whooley positive women (See Howard et al, 2018)[26] in all analyses apart from examining differences in socio-demographics between GAD-2 positives and GAD-2 negatives. The population prevalence of anxiety disorders and comorbid disorders were estimated based on weighted

For peer review only - http://bmjopen.bmj.com/site/about/guidelines.xhtml

diagnostic interview responses (using Stata's 'svy' command). Bootstrap re-sampling of the weighted estimators was used for calculation of confidence intervals.

As pre-specified in our analysis plan, the weighted rates of "true positive", "false positives", "true negatives" and "false negatives" were tabulated for GAD, any anxiety disorder (including PTSD & OCD) and any anxiety disorder (without PTSD or OCD). Using these values, the sensitivity, specificity, PPV, NPV and likelihood ratios (positive and negative) were calculated.

Missing data

Of the 545 participants, 24 (4%) women had some SCID missing data: one participant on GAD and all eating disorders; one participant on agoraphobia, specific phobia, and PTSD; one participant on mixed anxiety and depression, Borderline Personality disorder and PTSD; one participant on hypomanic, manic, current major depressive disorder and bipolar I & II; one participant on all eating disorders; and 19 participants on the PTSD module. List-wise deletion (performed in Stata) was used to calculate frequencies of SCID disorders in the study sample.

To calculate population prevalence of the SCID disorders, missing observations in the SCID items were accounted for by using inverse probability weights that incorporated the Whooley sampling, as well as variables that were significant in predicting missingness of SCID responses (for full details of weightings and analysis strategy see Howard et al, 2018).[26] Due to the large numbers of women who declined to respond to questions on the PTSD module, we carried out a sensitivity analysis for the prevalence estimate of PTSD in which we first assumed that all missing data were actually cases of PTSD, and then assumed that all missing data were not cases of PTSD.

Seventeen participants (3%) had missing GAD-2 data (15 for both questions, 1 for GAD-2 question 1 and 1 for GAD-2 question 2). No imputation was performed for women with missing data on GAD-2 items because missing data for one question would mean 50% data missing. These were therefore treated as missing observations and only women with complete data on both GAD-2 questions were used in the analyses to investigate sensitivity and specificity (n=528). Out of 528 women with complete data on the GAD-2 questions, there was missing data on the following SCID anxiety modules: one participant had missing data on PTSD, agoraphobia and specific phobia and 18 participants had missing data on the PTSD module.

For peer review only - http://bmjopen.bmj.com/site/about/guidelines.xhtml

Ethics approval: The research was approved by the National Research Ethics Service, London Committee - Camberwell St Giles (ref no 14/LO/0075). Participants were provided with study information sheets which was fully explained to them, had the opportunity to ask questions and gave informed consent prior to taking part the study. No adverse events occurred for women taking part in the study during the research interview. Where risk (e.g. significant suicidality, safeguarding issues) was identified during the research interview, researchers discussed this with the study PI and the woman's midwife and/or GP were informed, following consent to information sharing by the study participant (all women were aware this occurred and consented to this).

RESULTS

Sample characteristics

Between 10/11/14 and 30/06/16, 10004 women attended their initial antenatal booking appointment with a midwife at the study site. Of these women, 41 did not have Whooley answers recorded so the base population consisted of 9963 women. The total number of eligible women recruited into the WENDY study was 545 women. This sample was similar to the base population on sociodemographic factors such as age, ethnicity and number of children (see Howard, et al. [21]). Of the total number of women recruited into the WENDY study, 528 (97%) provided answers to the GAD-2 questions (see Figure 1). Sociodemographic characteristics (age, ethnicity and number of children) of women that provided GAD-2 answers were similar to the rest of the WENDY sample and wider base population (see online supplementary file 1). There were 119 (23%) GAD-2(≥3) positives and 409 (77%) GAD-2 (<3) negatives within our study sample, where GAD-2 (≥3) positive was defined as reporting a total score of 3 or more on the GAD-2 questions (see Table 1). Compared to GAD-2 negatives, GAD-2 positives were more likely to be single, have lower levels of education, be unemployed or not working due to illness, have a lower income, and have an unplanned pregnancy. When scoring for GAD-2 positives was defined as scoring 1 or more, there were 226 (43%) GAD-2 (Yes/No) positives and 302 (57%) GAD-2 (Yes/No) negatives within our study sample.

[INSERT TABLE 1 HERE]

For peer review only - http://bmjopen.bmj.com/site/about/guidelines.xhtml

SCID Anxiety disorder prevalence and comorbidity

Using weighted estimates, the population prevalence was estimated as 17% (95%CI: 12 to 21%) for any SCID anxiety disorder (or 15% (95%CI: 11 to 19%) for any SCID anxiety disorder excluding PTSD and OCD, as in DSM-V). Specifically, there was an estimated population prevalence of 5% (95%CI: 3 to 6%) for GAD, 4% (95%CI: 2 to 6%) for social phobia, 8% (95%CI: 5 to 11%) for specific phobia, 0.2% (95%CI: 0.03 to 0.3%) for panic disorder, 0.4% (95%CI: 0 to 2%) for agoraphobia, 2% (95%CI: 1 to 4%) for OCD, and 0.8% (95%CI: 0 to 1%) for PTSD. As missing data were particularly common for the PTSD module, a sensitivity analysis demonstrated that when all women with missing data are assumed to have PTSD, the population prevalence estimate was 4% (95%CI: 2 to 6%). When all women with missing data are assumed not to have PTSD, the population prevalence estimate was 0.8% (95%CI: 0.1 to 1.4%). Of note, of the women who declined to respond to the PTSD questions, 8 had already disclosed severe trauma earlier during the research interview which were related to being physically abused, sexual abuse/rape and witnessing violence.

The estimated population prevalence of comorbid depression with any anxiety disorder was 5% (95%CI: 2 to 7%) (or 4% (95%CI: 2 to 6%) for comorbid depression and any anxiety disorder excluding PTSD and OCD). Comorbid depression and GAD was estimated as 2% (95%CI: 1 to 3%). Please see Table 2 for unweighted count (%) and weighted estimates of population prevalence (%) of comorbidity between SCID depression and all anxiety disorders (including PTSD and OCD).

[INSERT TABLE 2 HERE]

Diagnostic accuracy of the GAD-2 screening questions in identifying GAD

SCID GAD was found in 23 (6%) of GAD-2 (<3) negative and 35 (29%) of GAD-2 (≥3) positive women. After adjusting for weights, GAD was estimated to occur in 137 (2%, 95%CI: 1 to 3%) of GAD-2 (<3) negatives and 302 (26%, 95%CI: 15 to 40%) of GAD-2 (≥3) positives. 8439 (98%, 95%CI: 97 to 99%) GAD-2 (<3) negatives and 872 (74%, 95%CI: 60 to 85%) GAD-2 (≥3) positives did not meet criteria for SCID GAD (see online supplementary files 2 & 3 for cross-tabulation and proportions). Weighted sensitivity was 0.69, specificity 0.91, PPV 0.26, NPV 0.98, LR+ 7.35 and LR- 0.34. When scoring of the GAD-2 was changed to ≥1 (Yes/No response), 58 (19%) of GAD-2 (Yes/No) positive women met criteria for SCID

For peer review only - http://bmjopen.bmj.com/site/about/guidelines.xhtml

GAD, whereas there were no GAD-2 (Yes/No) negative women that met criteria for SCID GAD. After adjusting for weights, SCID GAD was estimated in 439 (11%, 95%CI: 7 to 16%) of GAD-2 (Yes/No) positives. Whereas, 5633 (100%) of GAD-2 (Yes/No) negatives and 3678 (89%, 95%CI: 84 to 93%) GAD-2 (Yes/No) positives did not meet criteria for SCID GAD (see online supplementary files 2 & 4 for cross-tabulation and proportions). The weighted sensitivity was 1, specificity 0.60, PPV 0.11, NPV 1, LR+ 2.53 and LR- 0.

Diagnostic accuracy of the GAD-2 screening questions in identifying any anxiety disorder

SCID anxiety disorders (including GAD, PTSD and OCD) were found in 73 (18%) of GAD-2 (<3) negatives and 57 (50%) of GAD-2 (≥3) positive women. After adjusting for weights, an estimated 1125 (13%, 95%CI: 10 to 18%) of GAD-2 (<3) negatives and 404 (36%, 95%CI: 23 to 51%) of GAD-2 (≥3) positives met criteria for any SCID anxiety disorder (including PTSD and OCD). 7268 (87%, 95%CI: 82 to 90%) GAD-2 (<3) negatives and 723 (64%, 95%CI: 49 to 77%) GAD-2 (≥3) positives had no anxiety disorders (see online supplementary file 5 & 6 for cross-tabulation and proportions). Weighted sensitivity was 0.26, specificity 0.91, PPV 0.36, NPV 0.87, LR+ 2.92 and LR- 0.81. When scoring of the GAD-2 was changed to ≥1 (Yes/No response), 22 (10%) of GAD-2 (Yes/No) negative and 108 (37%) of GAD-2 (Yes/No) positive women met criteria for any anxiety disorder (including PTSD and OCD). After adjusting for weights, 453 (8%, 95%CI: 5 to 13%) of GAD-2 (Yes/No) negatives and 1076 (27%, 95%CI: 20 to 35%) of GAD-2 (Yes/No) positives met criteria for any SCID anxiety disorder (including PTSD and OCD). Whereas, 5110 (92%, 95%CI: 87 to 95%) GAD-2 (Yes/No) negatives and 2881 (73%, 95%CI: 65 to 80%) GAD-2 (Yes/No) positives had no anxiety disorders (see online supplementary files 5 & 7 for cross-tabulation and proportions). Weighted sensitivity was 0.70, specificity 0.64, PPV 0.27, NPV 0.92, LR+ 1.95 and LR- 0.46. The exclusion of PTSD and OCD to the group of “any” anxiety disorders made little difference (see Table 3 and online supplementary file 8 for cross-tabulation). Sensitivity analysis of when PTSD missing data were considered as cases of PTSD and not cases of PTSD also did not make much difference (see Table 3 and online supplementary file 9 for cross-tabulations).

[INSERT TABLE 3 HERE]

For peer review only - http://bmjopen.bmj.com/site/about/guidelines.xhtml

DISCUSSION

In this inner city maternity population, the population prevalence estimated for Generalised Anxiety Disorder (GAD) was 5% (95%CI: 3 to 6%) and for all anxiety disorders was 17% (95%CI: 12 to 21%), in line with other studies.[11] The population prevalence estimated for PTSD was lower (0.8%, 95%CI: 0 to 1%) than the mean prevalence of 3.86% reported in a recent systematic review of PTSD identified by interviews during pregnancy.[27] However, as some women in our study who declined to answer the PTSD module also reported severe trauma elsewhere during the research interview, we conducted a sensitivity analysis where missing data were assumed firstly to be cases of PTSD and then in a second analysis not to be PTSD cases: the prevalence estimate of PTSD was then potentially as high as 4% (95%CI: 2 to 6%). Thus, the low prevalence of PTSD in our main analysis may reflect barriers to disclosure of trauma and associated symptoms.

As others have reported,[28 29] comorbidity is also common and we found estimates for comorbid depression and GAD (2%, 95%CI: 1 to 3%) that were similar to previous estimates derived from representative samples and utilising diagnostic clinical interviews (GAD and depression 2% (95%CI: 0 to 3%).[28] However, the population prevalence estimated in our sample of comorbid depression and any anxiety (5%, 95%CI: 2 to 7%) was slightly lower than previous estimates (7%, 95%CI: 3 to 11).[28]

This study makes a novel contribution to the gap in the literature by formally examining the diagnostic accuracy of the GAD-2 screening questionnaire for women during early pregnancy. The GAD-2 (using a cut-off of ≥3; as recommended by NICE) had a reasonable likelihood ratio when used in early pregnancy for identifying generalised anxiety disorder (LR+: 7 i.e. above 5, which has been suggested to indicate a potentially useful tool in clinical practice)[30] . However, GAD is not very common and the positive predictive value is low (26%). Furthermore, the diagnostic accuracy of the GAD-2 for identifying other anxiety disorders was poor (LR+2.92). This evidence suggests that the GAD-2 is not a helpful tool for maternity services as it will generate many false positives.

There are several strengths to this study. Firstly, we recruited a stratified representative sample of women using language interpreters to facilitate inclusion of non-English speaking women (Howard et al, 2018).[21] Secondly, we used an efficient and robust sampling design which facilitated the estimation of population prevalence's and we used a gold standard diagnostic interview. Limitations include recruitment from a single maternity site in London, though the single South London maternity site

For peer review only - http://bmjopen.bmj.com/site/about/guidelines.xhtml

utilised in this study included a very ethnically and socioeconomically diverse population. There were some missing data (though this was rare other than for PTSD), and timing of administering the GAD-2 in early pregnancy may have over-inflated the risk of "false positives".

Implications

This study does not support the NICE recommendation to use the GAD-2 in early pregnancy, due to its low positive predictive value even for generalised anxiety disorder (when applying a cut-off of both ≥3 or ≥1 indicating a yes response) and low effectiveness for "any anxiety disorder". Its accuracy later in pregnancy warrants further study but a recent systematic review reported the prevalence of self-reported anxiety symptoms to increase through pregnancy (trimester 1: 18%, trimester 2: 19%, trimester 3: 25%); [11] diagnostic accuracy is therefore unlikely to improve.

Following the NICE CG192 guideline recommendations, some services have already implemented the GAD-2 in routine practice. The findings from the current paper challenge the use of expert consensus and extrapolation from evidence derived from the general population to pregnant women when making NICE recommendations specifically for pregnant women. We argue that recommendations for pregnant women should be evidence based and propose that, at present, only the NICE recommendation on use of the Whooley questions is supported by evidence.[21] Currently, the evidence suggests that implementation of routine use of the GAD-2 is unwarranted. This brings us to a number of potential unexplored future research directions, which include a comprehensive diagnostic accuracy study, including data on acceptability, of the full GAD-7 questionnaire in pregnancy and the three "anxiety" items in the Edinburgh Postnatal Depression Scale in pregnancy. For the GAD-7, currently there is no evidence. There is some, conflicting evidence for the three "anxiety items" in the Edinburgh Postnatal Depression Scale, but as others have suggested, research is needed to determine their validity, reliability and diagnostic accuracy as a measure of antenatal anxiety disorders.[31 32] Furthermore, use of other routinely collected data including the Whooley questions could be used in a model (using predictive modelling techniques) to identify anxiety disorders or perhaps even more importantly, "any mental disorder". Finally, further research is needed to examine ways to overcome the barriers to disclosure of trauma and trauma-related symptoms in pregnancy. This suggests that more sensitive methods need to be developed to ask women about trauma.

For peer review only - http://bmjopen.bmj.com/site/about/guidelines.xhtml

Conclusions

This study suggests that anxiety disorders are common in early pregnancy but the GAD-2 screening measure is not useful during early pregnancy. In addition, even if asked directly, PTSD may be particularly difficult to detect in routine practice (as some women may decline to answer specific questions in relation to symptoms arising from traumatic experiences) and therefore further research is needed on how to overcome barriers to disclosure of trauma related symptoms.

Acknowledgements: We are grateful for the advice received regarding the WENDY study from our Patient and Public Advisory Group, our Programme Steering Committee (Prof Rona McCandlish (Chair), Dr Heather O'Mahen, Dr Pauline Slade, Ceri Rose and Rosemary Jones) and our Data Monitoring and Ethics Committee (Roch Cantwell (chair), Liz McDonald-Clifford, Marian Knight, Stephen Bremner). We also want to take the opportunity to thank the women who participated in this study.

Author Contributions: LH, DB, JM & AP conceived, planned and obtained funding for the ESMI programme of research and they, with KT, wrote the first draft of the WENDY study protocol. JD was involved in modifying study protocol. SN was involved in the literature search, planning and carrying out statistical analysis, interpretation of data and drafted the manuscript. ER and AP provided guidance on statistical analysis. Both LH and ER were involved with interpretation of data and revision on manuscript drafts. All authors commented on subsequent drafts, interpretation of data, and approved final version of manuscript. Professor Louise Howard (senior author and chief investigator) is the guarantor of the study.

Funding: This paper summarises independent research funded by the National Institute for Health Research (NIHR) under the Programme Grants for Applied Research programme (ESMI Programme: grant reference number RP-PG-1210-12002) and the National Institute for Health Research (NIHR) / Wellcome Trust Kings Clinical Research Facility and the NIHR Biomedical Research Centre and Dementia Unit at South London and Maudsley NHS Foundation Trust and Kings College London. The views expressed are those of the author(s) and not necessarily those of the NHS, the NIHR or the Department of Health. The study team acknowledges the study delivery support given by the South London Clinical Research Network. The funder had no role in the design or conduct of the study; the collection, management, analysis, and interpretation of the data; or the writing of the article.

For peer review only

For peer review only - http://bmjopen.bmj.com/site/about/guidelines.xhtml

Competing interests: All authors have completed the ICMJE uniform disclosure form at www.icmje.org/coi_disclosure.pdf and declare support from the National Institute for health Research for the submitted work; no financial relationships with any organisations that might have an interest in the submitted work in the previous three years; no other relationships or activities that could appear to have influenced the submitted work. Louise Howard (senior author and chief investigator), chaired the NICE CG192 guidelines development group on antenatal and postnatal mental health in 2012-4. No other conflicts of interest.

Data sharing and access to study protocol: Full study protocol (approved by Research Ethics Committee) and patient level data is available from Chief Investigator Professor Louise Howard (louise.howard@kcl.ac.uk). Consent for data sharing was not obtained but the presented data are anonymised and risk of identification is very low.

For peer review only

TABLES

Table 1: Socio-demographics and characteristics of participants with GAD-2 data included in diagnostic accuracy analyses (n=528)

	GAD-2 Negative Score < 3 N=409	GAD-2 Positive Score ≥ 3 N=119	p	Overall (total) N=528
**Age (years)**			0.126	
16 – 19	4 (1%)	3 (3%)		7 (1%)
20-29	104 (25%)	40 (34%)		144 (27%)
30-39	267 (65%)	69 (58%)		336 (64%)
40+	34 (8%)	7 (6%)		41 (8%)
**Ethnicity**			0.384	
White	223 (55%)	56 (47%)		279 (53%)
Black/ Caribbean	123 (30%)	46 (39%)		169 (32%)
Asian/Asian British	19 (5%)	3 (3%)		22 (4%)
Mixed/Multiple ethnicity	17 (4%)	5 (4%)		22 (4%)
Other	27 (7%)	9 (8%)		36 (7%)
**Highest Education level**			0.006	
None/school qualifications	38 (9%)	24 (20%)		62 (12%)
College/Diploma/Higher/Certificate/training	147 (36%)	42 (35%)		189 (36%)
Degree level/ Postgraduate qualifications	224 (55%)	53(45%)		277 (52%)
**Employment status**[1]			0.010	
Employed	271 (66%)	69 (58%)		340 (65%)
Student	19 (5%)	2 (2%)		21 (4%)
Unemployed	42 (10%)	19 (16%)		61 (12%)
Homemaker	58 (14%)	14 (12%)		72 (14%)
Not working due to illness/Other	18 (4%)	14 (12%)		32 (6%)
**Income**[2]			<0.001	
< £15000	41 (13%)	32 (35%)		73 (18%)
£15,000-£30,999	54 (17%)	16 (17%)		70 (17%)
£31,000-£45,999	50 (16%)	10 (11%)		60 (15%)
£46,000-£60,999	52 (16%)	10 (11%)		62 (15%)
£61,000 or more	119 (38%)	24 (26%)		143 (35%)
**Relationship status**			<0.001	
Single	34 (8%)	23 (19%)		57 (11%)
Partner but not cohabiting	57 (14%)	22 (18%)		79 (15%)
Married/cohabiting	313 (77%)	70 (59%)		383 (72%)
Separated/divorced	5 (1%)	4 (3%)		9 (2%)
**Multiparous**				
No	202 (49%)	62 (52%)	0.603	264 (50%)
Yes	207 (51%)	57 (48%)		264 (50%)
**Planned pregnancy**				
Yes	280 (68%)	67 (56%)	0.014	347 (66%)
**Late booker**				
Yes	344 (84%)	94 (79%)	0.191	438 (83%)
**Translator required**				
Yes	25 (6%)	13 (11%)	0.074	38 (7%)

[1] 2 participants had missing data on employment status (1 GAD-2 positive and 1 GAD-2 negative)
[2] 120 participants had missing data on income (93 GAD-2 negatives, 27 GAD-2 positives)

For peer review only - http://bmjopen.bmj.com/site/about/guidelines.xhtml

Table 2: Co-morbidities in the WENDY sample including unweighted count (%) & *weighted population prevalence estimates % (95% CI)*

**Disorder**<br>**N**	Depression[1]<br>(n=545)	GAD<br>(n=544)	Panic disorder<br>(n=545)	Agoraphobia<br>(n=544)	Social phobia<br>(n=545)	Specific phobia<br>(n=544)	PTSD<br>(n=524)	OCD<br>(n=545)	Eating disorders[2]<br>(n=543)
Depression[1]<br>(n=545)	-	*2%*<br>*(1 - 3%)*	*0.2%*<br>*(0 - 2%)*	*0.06%*<br>*(0 – 0.3%)*[3]	*1%*<br>*(0 – 2%)*	*1%*<br>*(0 – 3%)*	*0.4%*<br>*(0 – 2%)*	*1%*<br>*(0 - 2%)*	*0.2%*<br>*(0-0.4%)*[3]
GAD<br>(n=544)	39/544 (7%)	-	*0.13%*<br>*(0.05 - 0.34%)*[3]	*0.35%*<br>*(0 - 2%)*[3]	*1%*<br>*(0 – 2%)*	*0.5%*<br>*(0 - 1%)*	*0%*	*0.1%*<br>*(0-0.3%)*[3]	*0.2%*<br>*(0-0.4%)*[3]
Panic disorder<br>(n=545)	5/545 (1%)	4/544 (1%)	-	*0%*	*0.03%*<br>*(0-0.2%)*[3]	*0%*	*0%*	*0.03%*<br>*(0-0.2%)*[3]	*0.03%*<br>*(0-0.2%)*[3]
Agoraphobia<br>(n=544)	2/544 (0.4%)	1/543 (0.2%)	0/544 (0%)	-	*0.4*<br>*(0.05-2%)*[3]	*0%*	*0%*	*0%*	*0%*
Social phobia<br>(n=545)	9/545 (1.7%)	6/544 (1%)	1/545 (0.2%)	1/544 (0.2%)	-	*1%*<br>*(0-2%)*	*0.07%*<br>*(0.02-0.3%)*[3]	*0.5%*<br>*(0 - 2%)*	*0.1%*<br>*(0.05-0.4)*[3]
Specific phobia<br>(n=544)	13/544 (2%)	6/543 (1%)	0/544 (0%)	0/544 (0%)	5/544 (1%)	-	*0%*	*1%*<br>*(0.2 – 3%)*[3]	*0.03%*<br>*(0 – 0.2%)*[3]
PTSD<br>(n=524)	11/524 (2%)	0/523 (0%)	0/524 (0%)	0/524 (0%)	2/524 (0.4%)	0/524 (0%)	-	*0.03%*<br>*(0 – 0.2%)*[3]	*0%*
OCD<br>(n=545)	11/545 (2%)	4/544 (1%)	1/545 (0.2%)	0/544 (0%)	5/545 (1%)	4/544 (1%)	1/524 (0.2%)	-	*0.08%*<br>*(0.02 – 0.3%)*[3]
Eating disorders[2]<br>(n=543)	4/543 (1%)	4/543 (1%)	1/543 (0.2%)	0/542 (0%)	4/543 (1%)	1/542 (0.2%)	0/522 (0%)	2/543 (0.4%)	-

[1] Depression includes all types of depression (major depressive disorder, mixed anxiety and depression).
[2] Eating disorders includes anorexia nervosa (including atypical), bulimia nervosa, binge eating disorder, purging disorder and other specified or eating disorder.
[3] Bootstrap CI could not be performed due to small sample size. Weighted CI are presented which take into account sampling and missing SCID.

For peer review only - http://bmjopen.bmj.com/site/about/guidelines.xhtml

Table 3: Sensitivity, specificity, PPV, NPV, LR+ and LR- of the GAD-2 questions (n=528)

GAD-2 cut-off	GAD	Any anxiety disorders (including PTSD and OCD)			Any anxiety disorders (excluding PTSD and OCD)[2]
		19 missing data on PTSD (n=516)[1]	If missing cases considered as PTSD cases	If missing cases considered as not PTSD cases	
Cut-off ≥ 3					
Sensitivity	0.69	0.26	0.26	0.26	0.26
Specificity	0.91	0.91	0.91	0.91	0.90
PPV	0.26	0.36	0.38	0.34	0.31
NPV	0.98	0.87	0.85	0.87	0.88
LR+	7.35	2.92	2.84	2.82	2.71
LR-	0.34	0.81	0.82	0.81	0.82
Cut-off ≥ 1 (Yes/No)					
Sensitivity	1	0.70	0.70	0.70	0.68
Specificity	0.60	0.64	0.64	0.63	0.62
PPV	0.11	0.27	0.30	0.26	0.23
NPV	1	0.92	0.91	0.92	0.92
LR+	2.53	1.95	1.95	1.90	1.81
LR-	0	0.46	0.47	0.47	0.51

[1] 19 participants had missing data on the PTSD module. 7 of these participants met criteria for other anxiety disorders. Therefore, the total sample size for any anxiety disorders was 516.

[2] We excluded PTSD and OCD to present the difference, as DSM-5 no longer considered PTSD and OCD as anxiety disorders.

For peer review only - http://bmjopen.bmj.com/site/about/guidelines.xhtml

FIGURE

Figure 1: Flow chart of women through the WENDY study during the study period (total recruited n=545) and women with available GAD-2 data (n=528)

REFERENCES

1. Kessler RC, Petukhova M, Sampson NA, et al. Twelve-month and lifetime prevalence and lifetime morbid risk of anxiety and mood disorders in the United States. *International journal of methods in psychiatric research* 2012;21(3):169-84.
2. Li SH, Graham BM. Why are women so vulnerable to anxiety, trauma-related and stress-related disorders? The potential role of sex hormones. *The Lancet Psychiatry* 2017;4(1):73-82.
3. Goodman JH, Chenausky KL, Freeman MP. Anxiety disorders during pregnancy: a systematic review. *The Journal of clinical psychiatry* 2014;75(10):1153-84.
4. Martini J, Petzoldt J, Einsle F, et al. Risk factors and course patterns of anxiety and depressive disorders during pregnancy and after delivery: A prospective-longitudinal study. *Journal of affective disorders* 2015;175:385-95.
5. Stein A, Pearson RM, Goodman SH, et al. Effects of perinatal mental disorders on the fetus and child. *The Lancet* 2014;384(9956):1800-19.
6. Glover V. Maternal depression, anxiety and stress during pregnancy and child outcome; what needs to be done. *Best practice & research Clinical obstetrics & gynaecology* 2014;28(1):25-35.
7. Newman L, Judd F, Komiti A. Developmental implications of maternal antenatal anxiety mechanisms and approaches to intervention. *Translational Developmental Psychiatry* 2017;5(1):1309879.
8. Barker ED, Jaffee SR, Uher R, et al. The contribution of prenatal and postnatal maternal anxiety and depression to child maladjustment. *Depress Anxiety* 2011;28(8):696-702. doi: 10.1002/da.20856 [published Online First: 2011/07/20]
9. Blair MM, Glynn LM, Sandman CA, et al. Prenatal maternal anxiety and early childhood temperament. *Stress* 2011;14(6):644-51. doi: 10.3109/10253890.2011.594121 [published Online First: 2011/07/28]
10. O'Connor TG, Heron J, Golding J, et al. Maternal antenatal anxiety and children's behavioural/emotional problems at 4 years. Report from the Avon Longitudinal Study of Parents and Children. *Br J Psychiatry* 2002;180:502-8.
11. Dennis C-L, Falah-Hassani K, Shiri R. Prevalence of antenatal and postnatal anxiety: systematic review and meta-analysis. *Br J Psychiatry* 2017;210(5):315-23. doi: 10.1192/bjp.bp.116.187179
12. National Institute for Health and Care Excellence. Antenatal and Postnatal Mental Health guidelines - Clinical guidelines CG192. London: National Institute for Health and Care Excellence (NICE), 2014.
13. Howard LM, Megnin-Viggars O, Symington I, et al. Antenatal and postnatal mental health: summary of updated NICE guidance. *BMJ* 2014;349:g7394.
14. Fontein-Kuipers Y, Nieuwenhuijze M, Ausems M, et al. Antenatal interventions to reduce maternal distress: a systematic review and meta-analysis of randomised trials. *BJOG: An International Journal of Obstetrics & Gynaecology* 2014;121(4):389-97.
15. Kelly RH, Danielson BH, Zatzick DF, et al. Chart-recorded psychiatric diagnoses in women giving birth in California in 1992. *Am J Psychiatry* 1999;156(6):955-57.
16. Darwin Z, McGowan L, Edozien LC. Antenatal mental health referrals: Review of local clinical practice and pregnant women ’ s experiences in England. *Midwifery* 2015;31(3):e17-e22.

For peer review only - http://bmjopen.bmj.com/site/about/guidelines.xhtml

17. Evans K, Spiby H, Morrell CJ. A psychometric systematic review of self-report instruments to identify anxiety in pregnancy. *Journal of advanced nursing* 2015;71(9):1986-2001.
18. Plummer F, Manea L, Trepel D, et al. Screening for anxiety disorders with the GAD-7 and GAD-2: a systematic review and diagnostic metaanalysis. *General Hospital Psychiatry* 2016;39:24-31.
19. First MB, Spitzer RL, Gibbon M, et al. Structured clinical interview for DSM-IV-TR axis I disorders, research version, patient edition. *New York: Biometrics Research, New York State Psychiatric Institute* 2002
20. Diagnostic A. statistical manual (DSM-V). *Washington, DC: American Psychiatric Association* 2013
21. Howard LM, Ryan EG, Trevillion K, et al. Accuracy of the Whooley questions and the Edinburgh Postnatal Depression Scale in identifying depression and other mental disorders in early pregnancy. *The British Journal of Psychiatry* 2018;212(1):50-56.
22. Spitzer RL, Kroenke K, Williams JB, et al. A brief measure for assessing generalized anxiety disorder: the GAD-7. *Archives of internal medicine* 2006;166(10):1092-97.
23. Kroenke K, Spitzer RL, Williams JBW, et al. Anxiety disorders in primary care: prevalence, impairment, comorbidity, and detection. *Ann Intern Med* 2007;146(5):317-25.
24. Celano CM, Suarez L, Mastromauro C, et al. Feasibility and utility of screening for depression and anxiety disorders in patients with cardiovascular disease. *Circulation: Cardiovascular Quality and Outcomes* 2013;6(4):498-504.
25. StataCorp. Stata Statistical Software: Release 15. College Station, TX: StataCorp LLC., 2017.
26. Howard L, Ryan E, Trevillion K, et al. The accuracy of the Whooley questions and the Edinburgh Postnatal Depression Scale in identifying depression and other mental disorders in early pregnancy. *The British Journal of Psychiatry* In Press
27. Yildiz P, Ayers S, Phillips L. The prevalence of posttraumatic stress disorder in pregnancy and after birth: A systematic review and meta-analysis. *Journal of Affect Disorders* 2017;208:634-45. doi: 10.1016/j.jad.2016.10.009
28. Falah-Hassani K, Shiri R, Dennis C-L. The prevalence of antenatal and postnatal co-morbid anxiety and depression: a meta-analysis. *Psychological Medicine* 2017:1-13.
29. Dindo L, Elmore A, O'Hara M, et al. The comorbidity of Axis I disorders in depressed pregnant women. *Archives of Women's Mental Health* 2017:1-8.
30. Grimes DA, Schulz KF. Refining clinical diagnosis with likelihood ratios. *The Lancet* 2005;365(9469):1500-05.
31. Matthey S, Fisher J, Rowe H. Using the Edinburgh postnatal depression scale to screen for anxiety disorders: Conceptual and methodological considerations. *Journal of Affective Disorders* 2013;146:224-30.
32. Milgrom J, Gemmill AW. Screening for perinatal depression. *Best Practice & Research Clinical Obstetrics & Gynaecology* 2014;28(1):13-23.

For peer review only - http://bmjopen.bmj.com/site/about/guidelines.xhtml

Figure 1: Flow chart of women through the WENDY study during the study period (total recruited n=545) and women with available GAD-2 data (n=528)

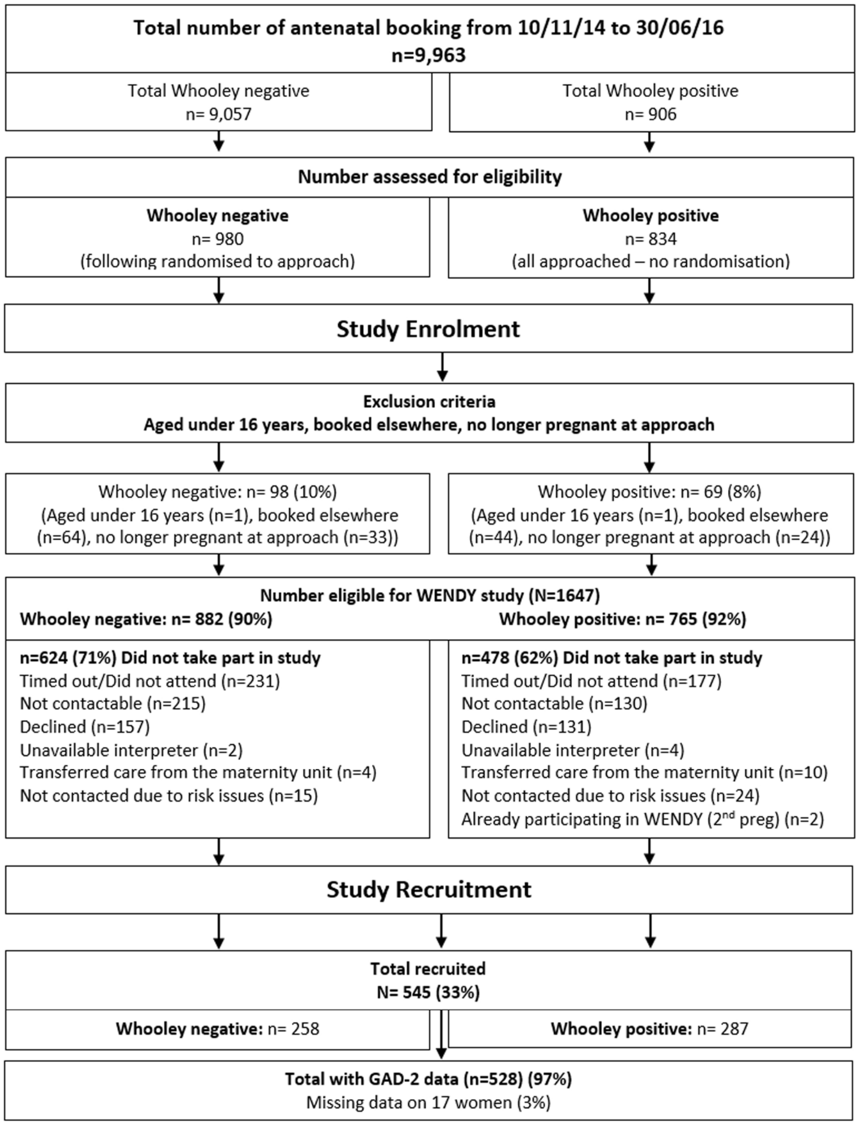

Figure 1: Flow chart of women through the WENDY study during the study period (total recruited n=545) and women with available GAD-2 data (n=528)

205x284mm (300 x 300 DPI)

Online supplementary file 1

Table showing characteristics of wider base population and study population

	Base population*	WENDY study sample N=545	Sample with GAD-2 data N=528
**Age (years)**	Mean: 31.67<br>Range: 14 – 52	Mean: 32.85<br>range: 16 – 47.5	Mean: 32.84<br>Range: 16 – 47.5
16 – 19	232 (2%)	8 (1%)	7 (1%)
20 – 29	3048 (30%)	150 (28%)	144 (27%)
30 – 39	6240 (61%)	341 (63%)	336 (64%)
40+	705 (7%)	46 (8%)	41 (8%)
**Ethnicity**			
White	4914 (51%)	284 (52%)	279 (53%)
Black/ Caribbean	3162 (33%)	177 (32%)	169 (32%)
Asian/Asian British	594 (6%)	25 (5%)	22 (4%)
Mixed/Multiple ethnicity	308 (3%)	23 (4%)	22 (4%)
Other	646 (7%)	36 (7%)	36 (7%)
**Other children**			
None	5077 (50%)	271 (50%)	264 (50%)
1	3209 (31%)	175 (32%)	167 (32%)
2 or more	1939 (19%)	99 (18%)	97 (18%)

*Base population covers women seen from 1.11.2014 - 30.6.2016, missing data not included.

For peer review only

Online supplementary file 2

Table of weighted population prevalence for calculations of sensitivity and specificity for GAD including count and proportion % (95% CI)

	**GAD-2 (<3) negative**	**GAD-2 (≥3) positive**
**No GAD**	8439<br>98% (97 – 99%)	872.2<br>74% (60 – 85%)
**SCID GAD**	136.5<br>2% (1 – 3%)	302.2<br>26% (15 – 40%)
	**GAD-2 (Yes/No) negative**	**GAD-2 (Yes/No) positive**
**No GAD**	5633<br>100%	3678<br>89% (84 – 93%)
**SCID GAD**	0<br>0%	438.7<br>11% (7 – 16%)

For peer review only

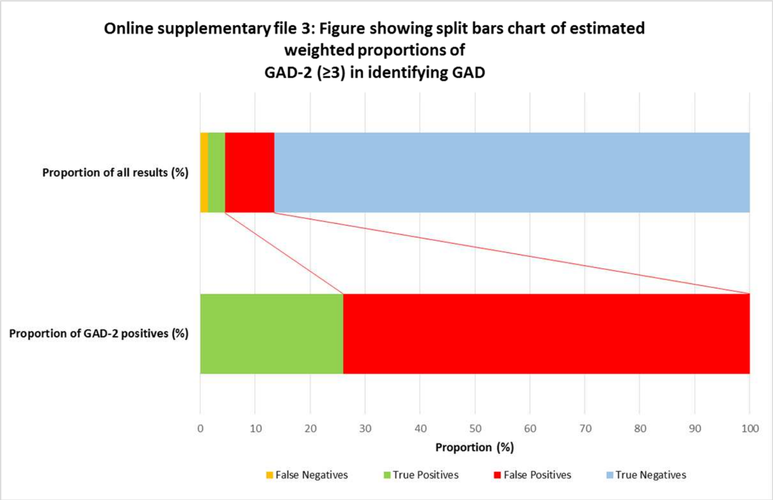

Online supplementary file 3: Figure showing split bars chart of estimated weighted proportions of GAD-2 (≥3) in identifying GAD

275x190mm (300 x 300 DPI)

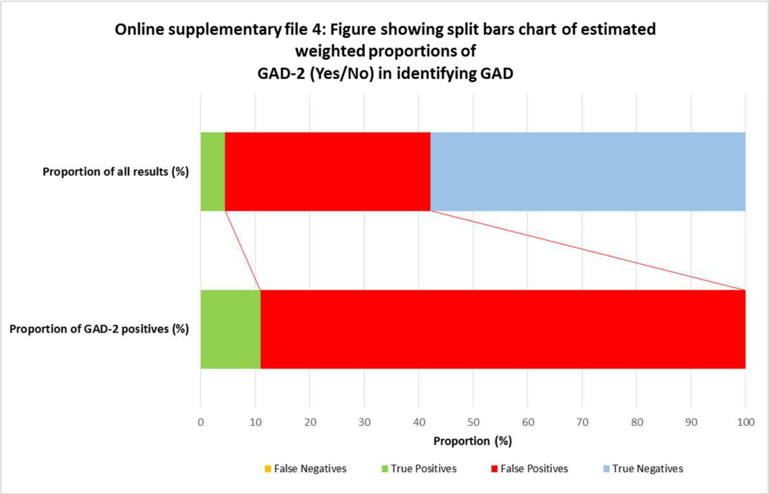

Online supplementary file 4: Figure showing split bars chart of estimated weighted proportions of GAD-2 (Yes/No) in identifying GAD

275x190mm (300 x 300 DPI)

Online supplementary file 5

Table of weighted population prevalence for calculations of sensitivity and specificity for any anxiety disorder (including PTSD and OCD) including count and proportion % (95% CI)

	**GAD-2 (<3) negative**	**GAD-2 (≥3) positive**
**No anxiety**	7268<br>87% (82 – 90%)	723.1<br>64% (49 – 77%)
**SCID any anxiety**	1125<br>13% (10 – 18%)	403.6<br>36% (23 – 51%)
	**GAD-2 (Yes/No) negative**	**GAD-2 (Yes/No) positive**
**No anxiety**	5110<br>92% (87 – 95%)	2881<br>73% (65 – 80%)
**SCID any anxiety**	452.8<br>8% (5 – 13%)	1076<br>27% (20 – 35%)

For peer review only

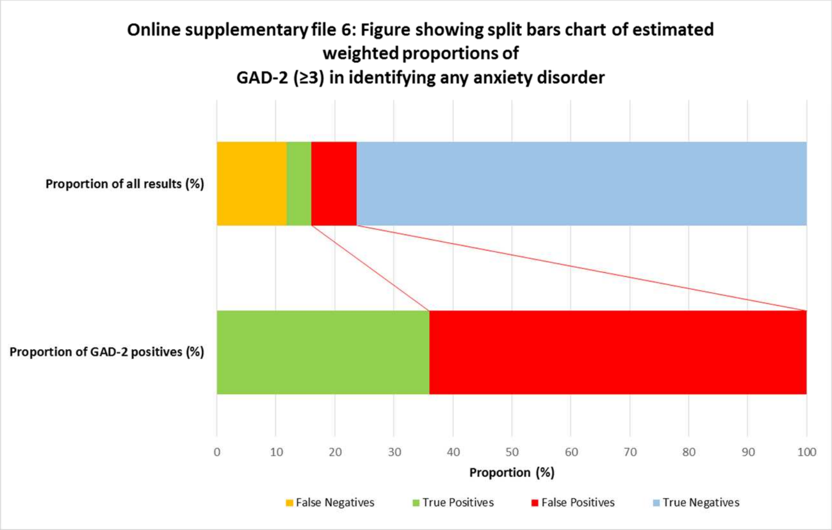

Online supplementary file 6: Figure showing split bars chart of estimated weighted proportions of GAD-2 (≥3) in identifying any anxiety disorder

254x190mm (300 x 300 DPI)

For peer review only - http://bmjopen.bmj.com/site/about/guidelines.xhtml

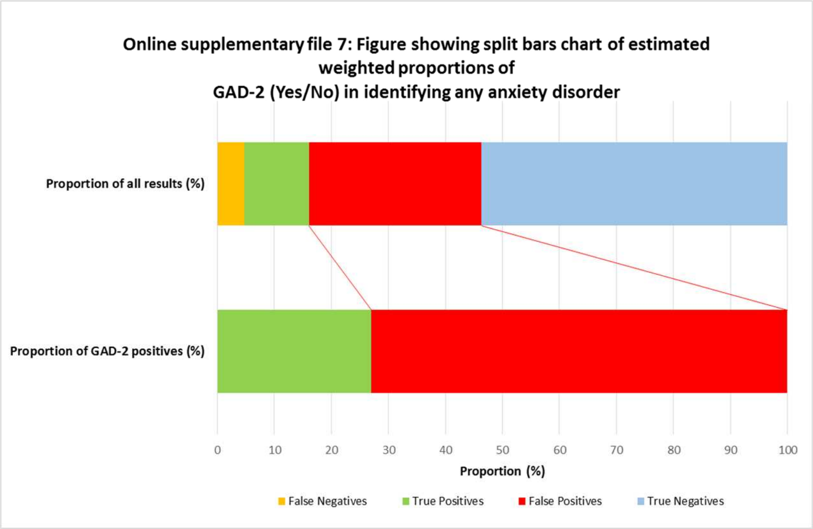

Online supplementary file 7: Figure showing split bars chart of estimated weighted proportions of GAD-2 (Yes/No) in identifying any anxiety disorder

254x190mm (300 x 300 DPI)

w only

Online supplementary file 8

Table of weighted population prevalence's for calculations of sensitivity and specificity for any anxiety disorder (excluding PTSD and OCD)

	GAD-2 (<3) negative	GAD-2 (≥3) positive
**No anxiety**	7536<br>88% (83 – 91%)	805.5<br>69 (54 – 80%)
**SCID any anxiety**	1039<br>12% (9 – 17%)	368.8<br>31 (20 – 46%)
	**GAD-2 (Yes/No) negative**	**GAD-2 (Yes/No) positive**
**No anxiety**	5187<br>92% (87 – 95%)	3155<br>77% (69 – 83%)
**SCID any anxiety**	446.5<br>8% (5 – 13%)	961.3<br>23% (17 – 31%)

For peer review only

Online supplementary file 9

Table of weighted population prevalence's for calculations of sensitivity and specificity for any anxiety disorder (including PTSD and OCD) (missing PTSD data considered as PTSD cases)

	**GAD-2 (<3) negative**	**GAD-2 (≥3) positive**
**No anxiety**	7268<br>85% (80 – 89%)	723.1<br>62% (47 – 74%)
**SCID any anxiety**	1307<br>15% (11 – 20%)	451.3<br>38% (26 – 53%)
	**GAD-2 (Yes/No) negative**	**GAD-2 (Yes/No) positive**
**No anxiety**	5110<br>91% (85 – 94%)	2881<br>70% (62 – 77%)
**SCID any anxiety**	523<br>9% (6 – 15%)	1235<br>30% (23 – 38%)

Table of weighted population prevalence's for calculations of sensitivity and specificity for any anxiety disorder (including PTSD and OCD) (missing PTSD data considered as not PTSD cases)

	**GAD-2 (<3) negative**	**GAD-2 (≥3) positive**
**No anxiety**	7450<br>87% (82 – 90%)	770.8<br>66% (51 – 78%)
**SCID any anxiety**	1125<br>13% (10 – 18%)	403.6<br>34% (22 – 49%)
	**GAD-2 (Yes/No) negative**	**GAD-2 (Yes/No) positive**
**No anxiety**	5181<br>92%(87 – 95%)	3040<br>74% (66 – 80%)
**SCID any anxiety**	452.8<br>8% (5 – 13%)	1076<br>26 (20 – 34%)

For peer review only - http://bmjopen.bmj.com/site/about/guidelines.xhtml

Section & Topic	No	Item	Reported on page #
**TITLE OR ABSTRACT**			
	**1**	Identification as a study of diagnostic accuracy using at least one measure of accuracy (such as sensitivity, specificity, predictive values, or AUC)	2
**ABSTRACT**			
	**2**	Structured summary of study design, methods, results, and conclusions (for specific guidance, see STARD for Abstracts)	2
**INTRODUCTION**			
	**3**	Scientific and clinical background, including the intended use and clinical role of the index test	4
	**4**	Study objectives and hypotheses	5
**METHODS**			
Study design	**5**	Whether data collection was planned before the index test and reference standard were performed (prospective study) or after (retrospective study)	6
Participants	**6**	Eligibility criteria	6 & figure 1
	**7**	On what basis potentially eligible participants were identified (such as symptoms, results from previous tests, inclusion in registry)	5, 6 & figure 1
	**8**	Where and when potentially eligible participants were identified (setting, location and dates)	5, 6 & figure 1
	**9**	Whether participants formed a consecutive, random or convenience series	5, 6 & figure 1
Test methods	**10a**	Index test, in sufficient detail to allow replication	6
	**10b**	Reference standard, in sufficient detail to allow replication	7
	**11**	Rationale for choosing the reference standard (if alternatives exist)	7
	**12a**	Definition of and rationale for test positivity cut-offs or result categories of the index test, distinguishing pre-specified from exploratory	6
	**12b**	Definition of and rationale for test positivity cut-offs or result categories of the reference standard, distinguishing pre-specified from exploratory	7
	**13a**	Whether clinical information and reference standard results were available to the performers/readers of the index test	6
	**13b**	Whether clinical information and index test results were available to the assessors of the reference standard	7
Analysis	**14**	Methods for estimating or comparing measures of diagnostic accuracy	8
	**15**	How indeterminate index test or reference standard results were handled	N/A
	**16**	How missing data on the index test and reference standard were handled	8
	**17**	Any analyses of variability in diagnostic accuracy, distinguishing pre-specified from exploratory	8
	**18**	Intended sample size and how it was determined	6 & Figure 1
**RESULTS**			
Participants	**19**	Flow of participants, using a diagram	Figure 1
	**20**	Baseline demographic and clinical characteristics of participants	9 & Table 1
	**21a**	Distribution of severity of disease in those with the target condition	10
	**21b**	Distribution of alternative diagnoses in those without the target condition	10
	**22**	Time interval and any clinical interventions between index test and reference standard	6
Test results	**23**	Cross tabulation of the index test results (or their distribution) by the results of the reference standard	10 & 11 (online supplementary files 2, 5, 8 and 9)
	**24**	Estimates of diagnostic accuracy and their precision (such as 95% confidence intervals)	10 & 11
	**25**	Any adverse events from performing the index test or the reference standard	9
**DISCUSSION**			
	**26**	Study limitations, including sources of potential bias, statistical uncertainty, and generalisability	12
	**27**	Implications for practice, including the intended use and clinical role of the index test	13
**OTHER INFORMATION**			
	**28**	Registration number and name of registry	2
	**29**	Where the full study protocol can be accessed	15
	**30**	Sources of funding and other support; role of funders	14

For peer review only

STARD 2015

AIM

STARD stands for "Standards for Reporting Diagnostic accuracy studies". This list of items was developed to contribute to the completeness and transparency of reporting of diagnostic accuracy studies. Authors can use the list to write informative study reports. Editors and peer-reviewers can use it to evaluate whether the information has been included in manuscripts submitted for publication.

EXPLANATION

A **diagnostic accuracy study** evaluates the ability of one or more medical tests to correctly classify study participants as having a **target condition.** This can be a disease, a disease stage, response or benefit from therapy, or an event or condition in the future. A medical test can be an imaging procedure, a laboratory test, elements from history and physical examination, a combination of these, or any other method for collecting information about the current health status of a patient.

The test whose accuracy is evaluated is called **index test.** A study can evaluate the accuracy of one or more index tests. Evaluating the ability of a medical test to correctly classify patients is typically done by comparing the distribution of the index test results with those of the **reference standard**. The reference standard is the best available method for establishing the presence or absence of the target condition. An accuracy study can rely on one or more reference standards.

If test results are categorized as either positive or negative, the cross tabulation of the index test results against those of the reference standard can be used to estimate the **sensitivity** of the index test (the proportion of participants *with* the target condition who have a positive index test), and its **specificity** (the proportion *without* the target condition who have a negative index test). From this cross tabulation (sometimes referred to as the contingency or "2x2" table), several other accuracy statistics can be estimated, such as the positive and negative **predictive values** of the test. Confidence intervals around estimates of accuracy can then be calculated to quantify the statistical **precision** of the measurements.

If the index test results can take more than two values, categorization of test results as positive or negative requires a **test positivity cut-off**. When multiple such cut-offs can be defined, authors can report a receiver operating characteristic (ROC) curve which graphically represents the combination of sensitivity and specificity for each possible test positivity cut-off. The **area under the ROC curve** informs in a single numerical value about the overall diagnostic accuracy of the index test.

The **intended use** of a medical test can be diagnosis, screening, staging, monitoring, surveillance, prediction or prognosis. The **clinical role** of a test explains its position relative to existing tests in the clinical pathway. A replacement test, for example, replaces an existing test. A triage test is used before an existing test; an add-on test is used after an existing test.

Besides diagnostic accuracy, several other outcomes and statistics may be relevant in the evaluation of medical tests. Medical tests can also be used to classify patients for purposes other than diagnosis, such as staging or prognosis. The STARD list was not explicitly developed for these other outcomes, statistics, and study types, although most STARD items would still apply.

DEVELOPMENT

This STARD list was released in 2015. The 30 items were identified by an international expert group of methodologists, researchers, and editors. The guiding principle in the development of STARD was to select items that, when reported, would help readers to judge the potential for bias in the study, to appraise the applicability of the study findings and the validity of conclusions and recommendations. The list represents an update of the first version, which was published in 2003.

More information can be found on http://www.equator-network.org/reporting-guidelines/stard.

For peer review only
